# Advances in the study of S100A9 in cardiovascular diseases

**DOI:** 10.1111/cpr.13636

**Published:** 2024-03-19

**Authors:** Fengling Chen, Ziyu He, Chengming Wang, Jiajia Si, Zhu Chen, Yuan Guo

**Affiliations:** ^1^ Hengyang Medical School University of South China Hengyang Hunan China; ^2^ Department of Cardiovascular Medicine, Zhuzhou Hospital Affiliated to Xiangya School of Medicine Central South University Zhuzhou Hunan China; ^3^ Hunan Key Laboratory of Biomedical Nanomaterials and Devices Hunan University of Technology Zhuzhou China

## Abstract

Cardiovascular disease (CVD) is a group of diseases that primarily affect the heart or blood vessels, with high disability and mortality rates, posing a serious threat to human health. The causative factors, pathogenesis, and characteristics of common CVD differ, but they all involve common pathological processes such as inflammation, oxidative stress, and fibrosis. S100A9 belongs to the S100 family of calcium‐binding proteins, which are mainly secreted by myeloid cells and bind to the Toll‐like receptor 4 and receptor for advanced glycation end products and is involved in regulating pathological processes such as inflammatory response, fibrosis, vascular calcification, and endothelial barrier function in CVD. The latest research has found that S100A9 is a key biomarker for diagnosing and predicting various CVD. Therefore, this article reviews the latest research progress on the diagnostic and predictive, and therapeutic value of S100A9 in inflammatory‐related CVD such as atherosclerosis, myocardial infarction, and arterial aneurysm and summarizes its molecular mechanisms in the progression of CVD, aiming to explore new predictive methods and to identify potential intervention targets for CVD in clinical practice.

## Graphical abstract

S100A9 is released by immune and tissue cells in response to stressors such as damage, inflammation, and oxidative stress. This leads to the amplification of inflammation, anti‐inflammatory effects, induction of endothelial dysfunction, promotion of vascular microcalcification, as well as stimulation of cell proliferation, autophagy, apoptosis, and death. S100A9 can serve as a biomarker for diagnosis, prediction, and evaluation of cardiovascular diseases, and it is also considered a potential therapeutic target.

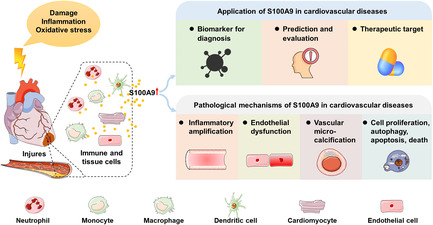

## 1 INTRODUCTION

Cardiovascular disease (CVD) is a global health problem that seriously endangers human health. The incidence of CVD in the globe continues to rise and is the leading cause of mortality.^1^ Recognized traditional risk factors for CVD comprise dyslipidaemia, diabetes, a familial predisposition to premature coronary heart disease, and tobacco use.^2^ However, the pathogenesis of CVD is complex, with its key pathological processes mainly involving activation of inflammatory response, oxidative stress, and pathological fibrosis, ultimately leading to damage to the heart and blood vessels.^3^, ^4^, ^5^, ^6^

Currently, inflammatory response is a crucial triggering factor in the occurrence and development of CVD.^7^ The elevated levels of inflammatory markers have been demonstrated to predict future cardiovascular events.^8^ During the inflammatory response, immune cells infiltrate the heart and blood vessels, releasing signals that attract additional immune cells and generate inflammatory mediators, leading to damage to the heart and blood vessels.^9^, ^10^, ^11^ This process also increases reactive oxygen species (ROS), which contributes to the growth of cardiac fibroblasts and the activation of matrix metalloproteinases, causing interstitial fibrosis in the heart.^11^ Furthermore, inflammation prompts cardiac fibroblasts to transform into myofibroblasts. The combined impact of inflammation and ROS results in the build‐up of extracellular matrix, causing interstitial fibrosis, ultimately leading to heightened stiffness in the heart and dysfunction of the myocardium.^9^, ^10^, ^11^

Furthermore, the inflammatory response of the vascular endothelium is closely related to CVD.^12^ Damaged and activated endothelial cells trigger leukocytes adhesion by releasing IL‐8, chemokines, adhesion molecules, and other cytokines. Infiltrating leukocytes induce endothelial cells to produce IL‐6 by releasing inflammatory cytokines such as TNF‐α, IL‐1β, promoting an endothelial pro‐inflammatory phenotypes, impairing endothelial function, and playing a crucial role in cardiovascular inflammation.^13^, ^14^, ^15^, ^16^ In summary, inflammation plays a significant regulatory role in the onset, development, and outcomes of CVD.^17^

S100A9, also known as calgranulin B or myeloid‐related protein‐14 (MRP‐14), predominantly functions as an inflammatory factor and is an endogenous damage‐associated molecular pattern molecule within the S100 calcium‐binding protein family, which is massively released under inflammatory conditions and plays a pivotal role in the progression of inflammation.^18^, ^19^, ^20^ Moreover, under physiological circumstances, S100A9 can non‐covalently bind with S100A8 to form a 24.5 kDa heterodimer, termed as calprotectin (S100A8/A9 or MRP‐8/14), which is present in vivo.^21^, ^22^ S100A9 and its heterodimer are effective ligands for the receptor for advanced glycation end products (RAGE) and Toll‐like receptor 4 (TLR4).^23^ But the affinity of S100A9 binding with RAGE and TLR4 is significantly higher than that of S100A8/A9, a phenomenon that might be associated with its biological function being regulated in intricate regulatory mechanisms.^23^

Upon release, S100A9 boosts the expression of inflammatory cytokines, chemokines, and fibrosis markers, while stimulating fibroblast proliferation, leading to the activation of inflammation response and tissue fibrosis.^24^, ^25^, ^26^, ^27^, ^28^ It has been reported that S100A9 can induce myocardial cell apoptosis through mediating ROS production and activating complement proteins C3 and C5 to produce anaphylatoxins C3a and C5a.^29^ Furthermore, S100A9 can activate the inflammatory response by modulating specific signalling pathways, which prompts disruption of endothelial barrier function. Subsequently, the disruption of endothelial function can amplify the inflammatory response and increase vascular permeability, thereby exacerbating vascular damage and instigating CVD.^30^, ^31^, ^32^, ^33^ These suggest that S100A9 is a key stimulator of CVD.

Thus, this review primarily focuses on elucidating the biological effects of S100A9 and its regulatory mechanisms in CVD. Specifically, considering the intimate association between CVD and inflammation, with S100A9 playing a prominent role as an inflammatory factor, we aim to summarize the intricate involvement of S100A9 in inflammation‐associated CVD, including atherosclerosis, myocardial infarction (MI), pulmonary arterial hypertension (PAH), aortic aneurysm, peripheral arterial disease (PAD), and so on (Table 1). We mainly elucidate the role of S100A9 in modulating inflammatory responses, amplifying tissue fibrosis, promoting vascular calcification, inducing endothelial dysfunction, and serving as a pivotal biomarker in inflammation‐associated CVD. This review is designed to furnish novel predictive indicator for CVD within clinical settings while exploring untapped therapeutic target.

**TABLE 1:** Evidence supporting the important role of S100A9 in cardiovascular disease.

	Diagnosis	Disease assessment	Treatment assessment	Prognosis
Atherosclerosis	Higher levels are found compared with healthy controls^31^, ^71^, ^72^ Higher levels are found in unstable plaques compared with stable ones^72^	A biomarker for rupture‐prone plaques^73^	Inhibition or knockout of S100A9 can alleviate atherosclerosis^63^, ^71^, ^74^, ^76^	High levels are predictive of disease progression^73^
Myocardial infarction	Higher levels are found compared with healthy controls or patients with stable angina^86^, ^87^, ^88^	A biomarker for predicting the onset of STEMI^85^	Early blockade of S100A9 is beneficial for cardiac repair, while prolonging the blockade may have adverse effects^78^, ^91^	High levels in the inflammatory phase may indicate poor prognosis^19^, ^80^, ^91^, ^92^ S100A9 mediates myocardial repair in the proliferative phase after MI^78^
Pulmonary arterial hypertension	Higher levels are found compared with healthy controls^104^	NA	NA	High levels predict an increased risk of PAH^109^
Preeclampsia	Higher levels are found compared with healthy controls^114^	NA	Administration of exogenous S100A9 induces preeclampsia^114^	NA
Hypertension	Higher levels are found compared with healthy controls^24^	NA	NA	Potential indicator for monitoring the occurrence and progression of hypertension^24^
Aneurysm	Higher levels are found compared with healthy controls^115^, ^117^ Higher levels are found in ruptured aneurysms compared with unruptured aneurysms^116^, ^117^ Higher levels are found early stage ruptured aneurysms compared with late‐stage ruptured aneurysms^116^	A biomarker for distinguishing aneurysms prone to rupture^116^, ^117^	NA	Positive correlation with prognosis^119^
Peripheral arterial disease	Higher levels are found compared with healthy controls^121^	A biomarker for predicting the severity of PAD^122^	Targeting S100A9 may be beneficial for restoring blood flow^125^	Positive correlation with prognosis^122^, ^123^
Hypertrophic cardiomyopathy	Higher levels are found compared with healthy controls^132^	NA	NA	NA
Uremic cardiomyopathy	Higher levels are found compared with healthy controls^130^	NA	Knockout of S100A9 improves cardiomyocyte hypertrophy and fibrosis^130^	NA
Atrial fibrillation	Higher levels are found compared with healthy controls^133^	NA	NA	Positive correlation with occurrence of AF^134^
Myocarditis	Higher levels are found compared with healthy controls^62^, ^137^	NA	Knockout of S100A9 attenuates myocardial inflammation^62^	Low S100A9 may suggest a favourable prognosis for myocarditis^136^
Infective endocarditis	NA	A crucial role in the inflammation and immune response of IE^138^	NA	NA

## 2 INTRODUCTION TO S100A9

S100A9 is a calcium‐ and zinc‐binding protein consisting of 114 amino acids with a molecular weight of approximately 13 kDa.^34^, ^35^ It possesses two EF‐hand domains, which form a highly conserved helix–loop–helix structure.^35^ And the gene encoding S100A9 is located on chromosome 1q21.3.^36^ S100A9 is mainly expressed in the immune system such as spleen, bone marrow, lymph nodes, lung and skin.^37^ S100A9 is predominantly located in the cytoplasm; however, in response to elevated intracellular calcium levels, it can translocate to the cytoskeleton and cell membrane or be secreted extracellularly.^18^, ^38^

S100A9 levels exhibit a notable elevation in pathological states, with the precise mechanism underlying its release remaining incompletely elucidated. However, research has shown that S100A9 release is regulated by E‐selectin.^39^ Specifically, E‐selectin mediates the formation of N‐terminal gasdermin D pores in neutrophils in an NLRP3‐dependent manner, leading to the release of S100A9.^39^ S100A9 is also regulated by p53.^29^ P53 is activated by the release of isoproterenol‐inducesd ROS, further upregulating the transcription of the S100A9 gene.^29^ An increase in S100A9 levels triggers an inflammatory response, which in turn promotes the release of ROS, forming a ROS/p53/S100A9 positive feedback loop.^29^

**FIGURE 1:**
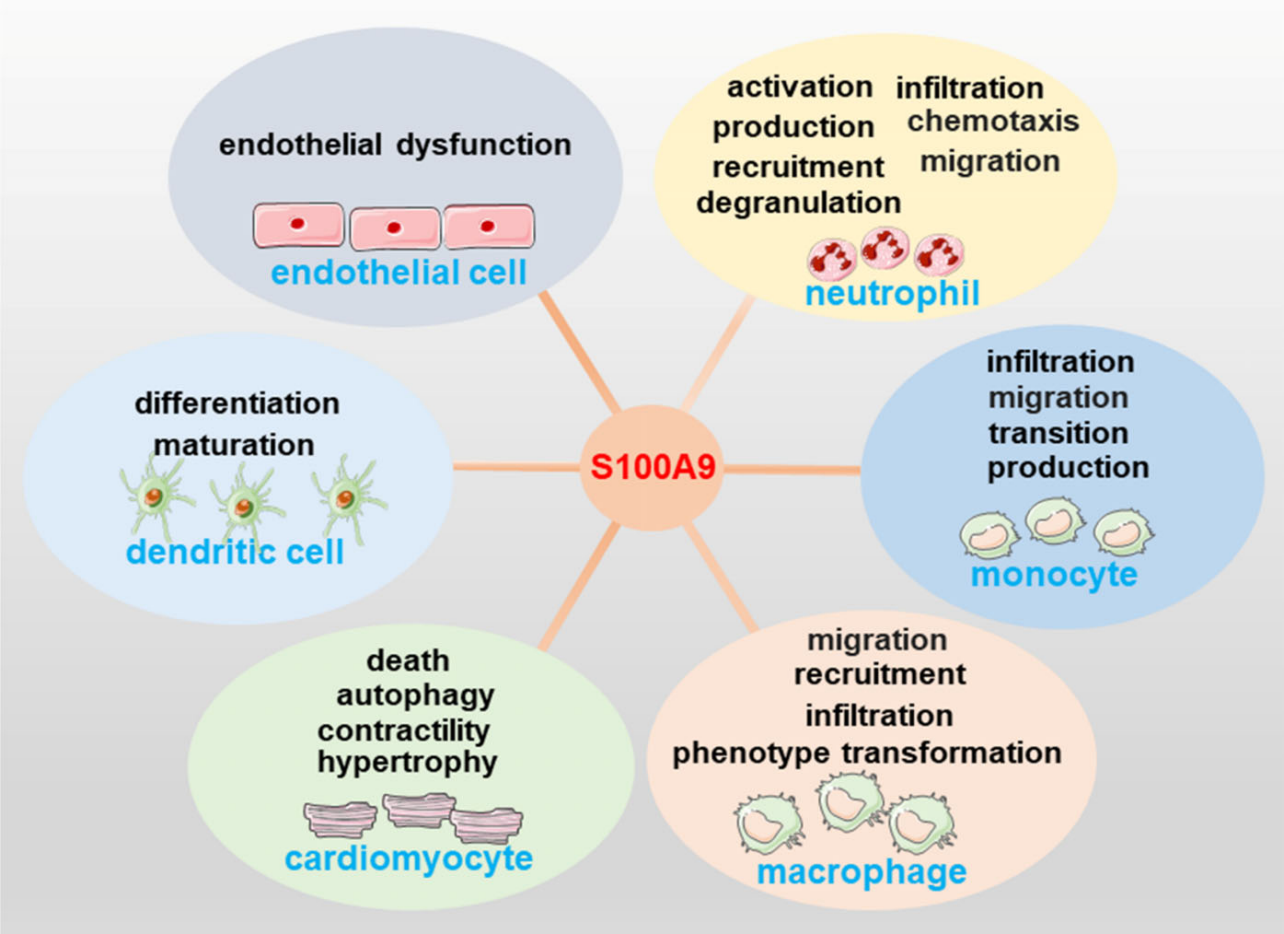
S100A9's influence on immune cell dynamics and cardiovascular function. S100A9 plays a pivotal role in modulating various aspects of immune cell behaviour and cardiovascular function. It regulates neutrophil infiltration, chemotaxis, migration, and recruitment, while also stimulating neutrophil production, activation, and degranulation. Additionally, S100A9 stimulates monocyte production, modulates monocyte infiltration, and induces monocyte migration, thereby indirectly influencing the transition from monocyte to macrophage. Moreover, S100A9 regulates macrophage phenotype transformation and infiltration, increases macrophage recruitment, and induces macrophage migration. Conversely, S100A9 inhibits dendritic cell differentiation and disrupts dendritic cell maturation. Furthermore, S100A9 promotes endothelial dysfunction and exerts detrimental effects on cardiomyocytes by inducing cardiomyocyte death, reducing contractility, suppressing autophagy, and inhibiting norepinephrine‐induced myocyte hypertrophy.

Under physiological conditions, S100A9 is generally underexpressed, primarily found in immune cells such as neutrophils.^40^ While under pathological conditions, S100A9 is released extracellularly by immune cells such as neutrophils, monocytes, macrophages, and dendritic cells, as well as necrotic myocardial cells, endothelial cells, and cancer cells, thereby performing corresponding functions such as modulating immune responses^27^, ^41^, ^42^, ^43^, ^44^, ^45^ (Figure 1). Interestingly, the expression levels of S100A9 in aged male mice are higher than those in young mice in all organs, a phenomenon possibly related to age‐associated inflammation.^46^

S100A9 exhibits a dual regulatory role in inflammatory responses. Under various stress stimuli, the substantial increase in S100A9 levels subsequently promotes the activation and migration of leukocytes, leading to a substantial recruitment of leukocytes to the site of inflammatory injury.^47^, ^48^ These recruited leukocytes, in turn, secrete various pro‐inflammatory cytokines, ROS, and other substances, thereby initiating a detrimental cycle of inflammation.^47^, ^48^ However, in abnormal systemic inflammation, S100A9 can exhibit an anti‐inflammatory action to avoid tissue damage caused by overwhelming inflammation.^49^ Other studies suggest that S100A8/A9 heterodimer and S100A8/S100A9‐tetramers also exert anti‐inflammatory effects, in the context of MI, psoriasis and arthritis lesions.^50^, ^51^ Furthermore, S100A9 has implicated in regulating cell proliferation, migration, and invasion in the context of chronic inflammation associated with cancer.^52^, ^53^

Besides regulating inflammation, S100A9 also plays a vital role in oxidative stress. In the context of oxidative stress, the expression of S100A9 significantly increases, leading to the activation of complement proteins C3 and C5 and subsequent myocardial injury.^29^ Li et al.^54^ confirmed that S100A9 can significantly induce cell apoptosis. When transiently transfected pEGFPC1‐S100A9 into p53^−/−^ and p53^+/+^ cells respectively, p53^+/+^ cells exhibited more severe apoptosis, suggesting that S100A9 induces apoptosis in a p53‐dependent manner.^54^ Further, Boteanu et al.^47^ found that inhibiting S100A9 can increase the expression of proteins related to the apoptosis process, such as NOL3, SOD2, and BAG3, thereby reducing cardiac oxidative stress and inhibiting pro‐apoptotic pathways. Overall, S100A9 plays a prominent role in regulating cell apoptosis under conditions of oxidative stress.

**FIGURE 2:**
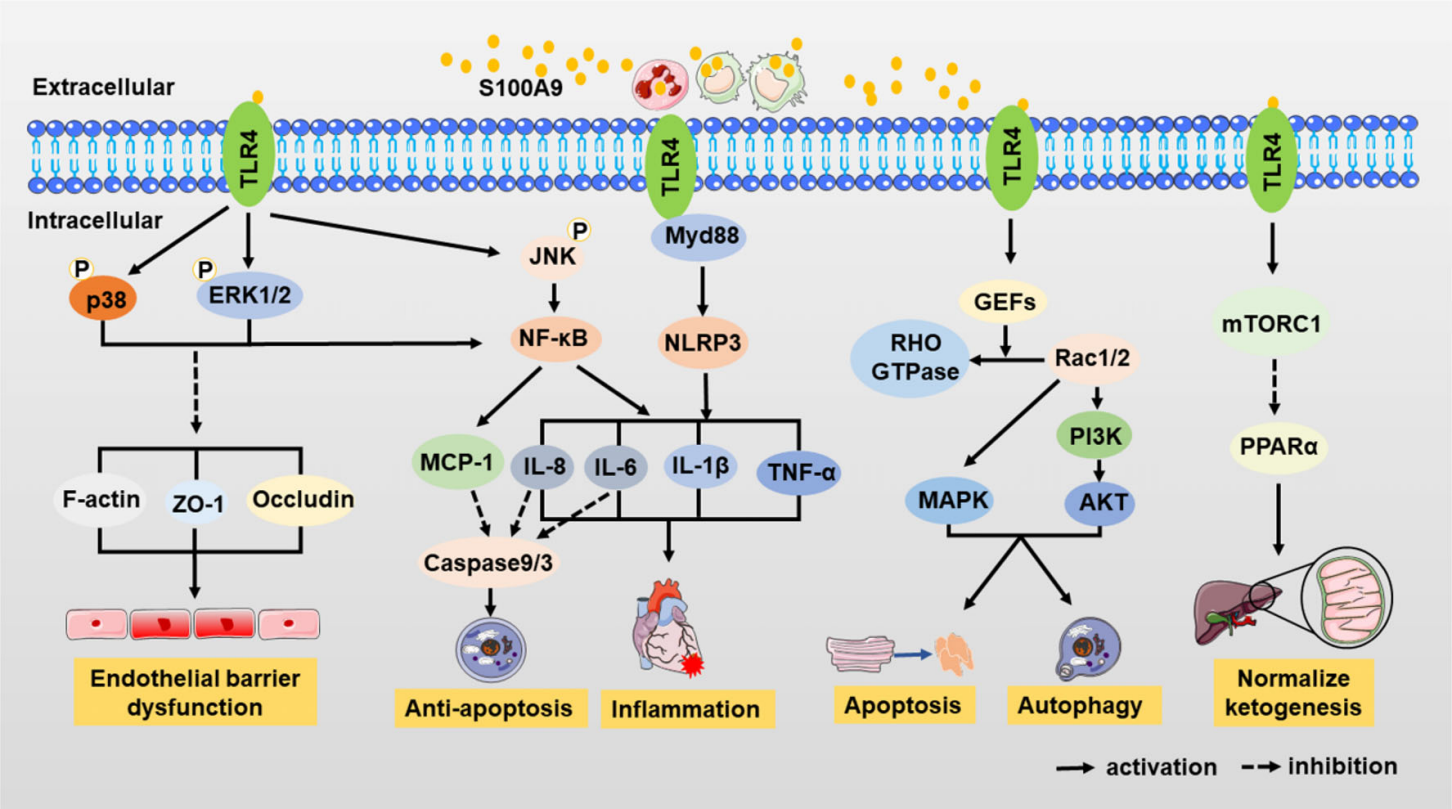
S100A9 binds to Toll‐like receptor 4 (TLR4) to activate downstream pathways for biological effects. S100A9 binds to TLR4, activating the MAPK pathway and subsequently NF‐κB, resulting in pro‐inflammatory cytokines release and pro‐inflammatory effects. S100A9 induces NLRP3 inflammasome activation via TLR4‐Myd88 and releases IL‐1β, leading to an exacerbation of inflammation. MAPK activation disrupts endothelial barrier function by breakdown F‐actin, ZO‐1, and occludin. Simultaneously, NF‐κB activation induces cytokine secretion and inhibits apoptosis through caspase 9/3 downregulation. S100A9‐TLR4 promotes apoptosis and autophagy through Rac1/2 activation of MAPK and PI3K‐AKT pathways. Additionally, their interaction normalizes ketogenesis by activating mTORC1 and inhibiting PPARα. p38, p38 mitogen‐activated protein kinase; ERK1/2, extracellular regulated protein kinases1/2; JNK, jun N‐terminal kinase; NF‐κB, nuclear factor‐κB; NLRP3, Nod‐like‐receptor family pyrin domain‐containing 3; Myd88, Myeloid differentiation factor 88; IL‐1β, interleukin‐1β; IL‐6, interleukin‐6; TNF‐α, tumour necrosis factor α; IL‐8, interleukin‐8; MAPK, mitogen‐activated protein kinases; MCP‐1, monocyte chemotactic protein‐1; GEFs, GMP exchange factors; PI3K, phosphoinositide 3‐Kinase; mTROC1, mammalian target of rapamycin C1; PPARα, peroxisome proliferator‐activated receptor‐alpha; ZO‐1, zonula occludens protein 1.

S100A9 possesses multiple receptors, with TLR4 and RAGE being the most extensively studied. Upon binding to its classic receptor TLR4, S100A9 induces the phosphorylation of p38, ERK1/2, and JNK, subsequently activating NF‐κB (Figure 2). This activation leads to the release of pro‐inflammatory cytokines, such as IL‐1β, IL‐6, TNF‐α, and IL‐8, thus exerting potent pro‐inflammatory effects.^25^, ^55^, ^56^ S100A9 induces NLRP3 inflammasome activation via TLR4‐Myd88 and releases IL‐1β, the latter interacts with the IL‐1R on myeloid progenitors to stimulate monocytes and neutrophils production, leading to an exacerbation of inflammation.^57^ Interestingly, although TLR4 is generally considered pro‐inflammatory, its interaction with S100A9 can normalize ketogenesis in diabetic mice by activating mTORC1, exhibiting anti‐inflammatory effects.^58^

Moreover, TLR4 induces the disassembly of F‐actin, ZO‐1, and occludin by activating downstream p38 and ERK1/2, significantly increasing endothelial cell permeability and disrupting endothelial barrier function, leading to tissue edema.^30^, ^59^ The activated S100A9/TLR4/MAPK/NF‐κB signalling pathway can also reduce caspase 9 and caspase 3 activity by inducing monocytes to secrete cytokines MCP‐1, IL‐6, and IL‐8 which act on cytokine receptors on the surface of neutrophils, thereby inhibiting cell apoptosis.^60^ In the early stages of MI, some S100A9 enters myocardial cells or other heart cells via TLR4 and combines with GMP exchange factors, which transforms Rac1/2 into activated Rho GTPases, and then Rac1/2 activates the MAPK signal pathway and PI3K‐AKT signal pathway, thereby directly regulating cell survival.^61^ Both signalling pathways can also indirectly govern autophagy and apoptosis via the mTOR signal pathway.^61^

**FIGURE 3:**
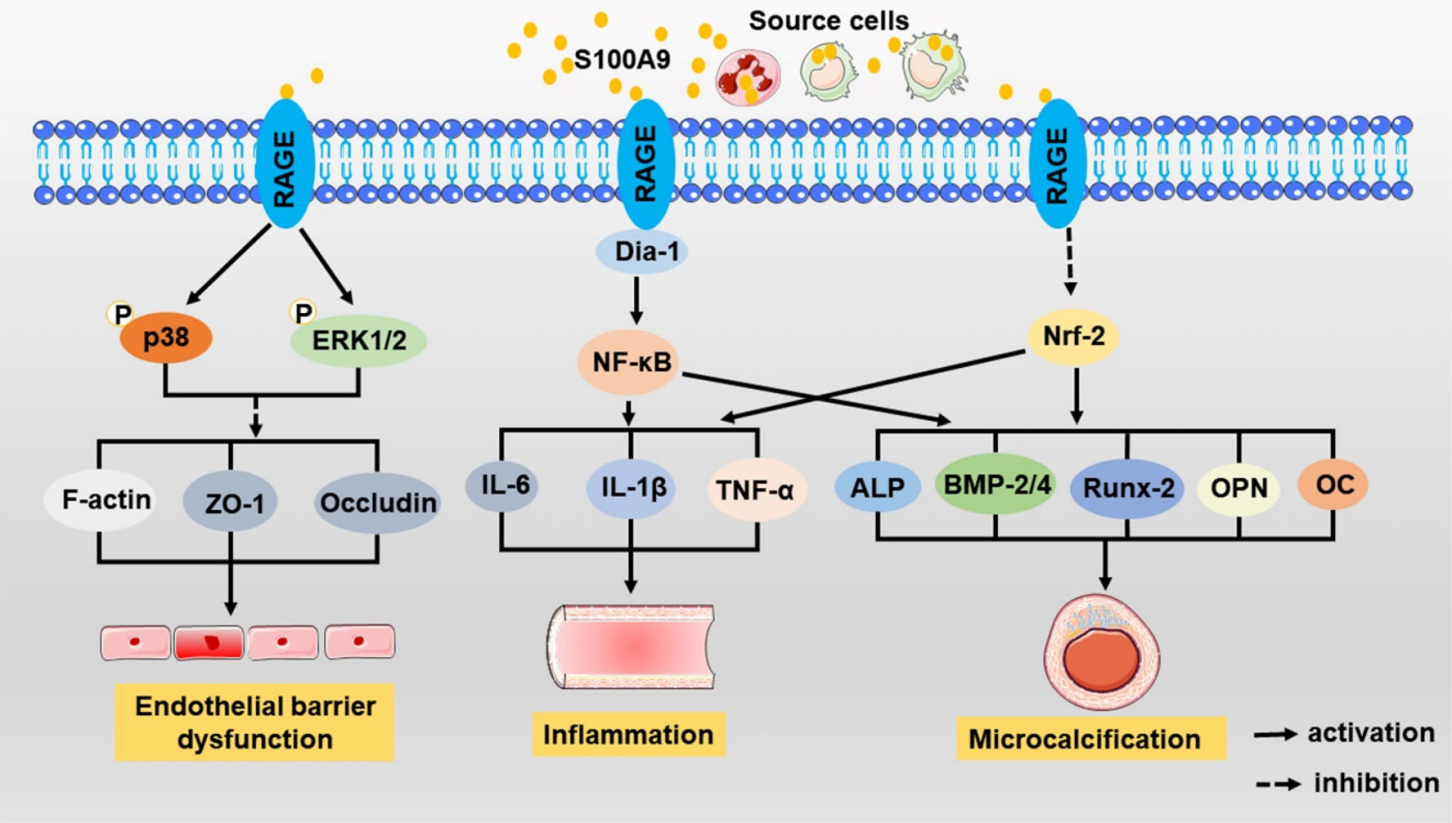
Role of the S100A9‐receptor for advanced glycation end products (RAGE) signalling pathway in cardiovascular disease. S100A9 binds to RAGE, activating NF‐κB and increasing pro‐inflammatory cytokines, leading to inflammation. It also activates p38 and ERK1/2, disrupting endothelial barrier function. Additionally, it decreases Nrf‐2 activity, promoting microcalcification of extracellular vesicles and regulating calcium homeostasis. Dia‐1, diaphanous‐1; Nrf‐2, nuclear factor 2 erythroid related factor 2; BMP‐2/4, bone morphogenetic protein 2/4; ALP, alkaline phosphatase; Runx‐2, runt‐related transcription factor 2; OC, osteocalcin; OPN, osteopontin.

Another classic receptor of S100A9 is RAGE. S100A9 binds to RAGE on the cell membrane surface, which further binds to its adaptor protein Dia‐1, activating NF‐κB and leading to an increase in the expression of inflammatory factors such as IL‐1β, IL‐6, and TNF‐α, thereby activating a systemic inflammatory response.^62^ It is worth mentioning that similar to TLR4, RAGE can also induce endothelial dysfunction via F‐actin, ZO‐1, and occludin.^30^, ^59^ The S100A9‐RAGE axis, activated by high glucose levels, induces an upregulation of NF‐κB activity and a downregulation of Nrf‐2 activity.^63^ This heightened NF‐κB activity leads to elevated expression of pro‐inflammatory cytokines from macrophages, while the reduced Nrf‐2 activity results in an increase in the expression of calcification‐promoting factors, such as osteocalcin, osteopontin, BMP‐2/4, ALP, and Runx‐2, in macrophage‐derived extracellular vesicles.^63^ This process promotes microcalcification of extracellular vesicles, thereby playing a role in regulating calcium homeostasis and contributing to vascular calcification, which links inflammation to microcalcification.^63^ The collective findings highlight the pivotal role of the S100A9‐RAGE axis in CVD, as it exhibits pro‐inflammatory properties, induces endothelial dysfunction, and contributes to vascular calcification (Figure 3).

However, it remains unclear whether S100A9 has independent actions beyond the aforementioned receptors. A study has demonstrated that S100A9 expressed by testicular macrophages activates the PI3K‐AKT signalling pathway, thus maintaining the M2 macrophages phenotype, which is associated with their immunosuppressive function.^40^ S100A9 also exerts a role in promoting cancer cell proliferation, migration, invasion, and recurrence. These effects are closely associated with the activation of ALDH1A1/Retinoic Acid signal pathway and Wnt/β‐catenin signal pathway. Activation of these pathways may result in immune evasion of cancer cells, ultimately contributing to tumour exacerbation.^64^, ^65^ Further investigation is needed to determine whether S100A9 directly activates these pathways or indirectly through TLR4 and RAGE exerts its effects.

## 3 ROLE OF S100A9 IN ATHEROSCLEROSIS

Atherosclerosis is currently conceived as a chronic inflammatory disease of the large‐medium sized arteries, triggered by traditional risk factors such as hyperlipidaemia and interactions between arterial wall cells and immune cells, which is characterized by lipid accumulation within the vascular wall, cell death, and chronic inflammation.^66^, ^67^, ^68^ Both innate immune responses and adaptive immune responses have been found to be activated in atherosclerosis.^69^, ^70^ Specifically, innate immune cells intake lipids via scavenger receptors or TLRs, triggering intracellular signalling cascades, which results in a series of gene expressions encoding pro‐inflammatory factors, thus inducing vascular inflammatory responses.^69^ Meanwhile, the induction of adaptive immune responses by T lymphocytes and B lymphocytes further exacerbates atherosclerosis.^70^

Studies have indicated that S100A9, as an inflammatory mediator, is noticeably increased in atherosclerosis and contributes to its development.^31^, ^71^, ^72^ By quantifying the concentration of the S100A9 protein in carotid plaque samples from 186 patients and analysing the proportion of S100A9‐expressing macrophages, Ionita et al.^72^ found that S100A9 and S100A9‐positive macrophages were highly expressed in unstable plaques compared with stable ones and correlated strongly with the pathological features and inflammatory status of unstable plaques. This suggests that S100A9 is a biomarker for rupture‐prone plaques.^72^ Langley et al.^73^ further performed a thorough proteomic analysis of the extracellular matrix in the plaques of atherosclerosis patients and identified elevated levels of S100A9 as potential biomarkers for atherosclerosis; further validation via the Bruneck study confirmed a positive correlation between high levels of S100A9 and the progression of atherosclerosis, the incidence of cardiovascular and cerebrovascular accidents during 10‐year follow‐up, suggesting S100A9 is a valuable biomarker for assessing the risk of unstable atherosclerosis, highlighting its importance in indicating atherosclerotic conditions.

Independent risk factor for atherosclerosis appears to include transient intermittent hyperglycaemia (TIH). This risk factor exerts its influence by stimulating extramedullary myelopoiesis, resulting in an elevated abundance of circulating inflammatory cells, such as Ly6C^hi^ monocytes and neutrophils, which accelerates atherogenesis.^74^ Flynn et al.^74^ reported that compared to mice transplanted with WT bone marrow, mice receiving S100A9^−/−^ bone marrow were shielded from myelopoiesis spurred on by TIH, leading to a reduction in circulating inflammatory cells, and ultimately attenuating atherosclerosis. This phenomenon may occur due to the blockade of the ROS‐S100A9‐RAGE axis induced by high blood glucose.^74^ Moreover, Hanssen et al.^75^ constructed a mouse model to mimic TIH by intravenously injection with MGO (a reactive glucose metabolite), which increased atherosclerotic burden via induces circulating neutrophils and monocytes, with an increase of S100A9 and RAGE. This indicates the activation of the S100A9‐RAGE axis by TIH leading to elevated levels of inflammatory factors, ultimately contributing to atherosclerosis.^75^ These studies collectively provide evidence that S100A9 is a key regulatory factor in atherogenesis induced by hyperglycaemia.

Furthermore, diabetes can hasten the development of vascular calcification, serving as a significant risk factor for atherosclerosis.^63^ Kraakman et al.^76^ have reported that the inhibition of S100A9 can reduce the formation of atherosclerosis in diabetic mice; in diabetic patients, inhibition of S100A9 bioactivity suppresses platelet production, which may help to reduce the incidence and severity of cardiovascular events. In plasma of fat‐fed ApoE^−/−^ mice, quantified levels of matrix vesicles were found to exhibit an upward trend compared to fat‐fed WT mice, while the level of matrix vesicles in plasma of ApoE^−/‐^S100A9^−/−^ mice fell to those of WT mice.^71^ This suggests that S100A9 may be a critical mediator of microcalcification in atherosclerosis.^71^ Kawakami et al.^63^ further validated the influence of macrophage‐derived S100A9 on atherosclerosis and discovered that control mice had S100A9‐positive macrophages in plaques, while almost none were found in plaques of siS100A9 mice, and the expression of inflammatory factors and vascular calcification markers were significantly reduced in splenic macrophages; in addition, colocalization of S100A9 with RAGE was observed in plaques. These findings confirm the involvement of the S100A9‐RAGE axis in macrophage‐mediated matrix vesicle microcalcification in diabetes, suggesting inhibition of this axis may alleviate vascular calcification and inflammation, and thus ameliorate the progression of atherosclerosis.^63^

## 4 ROLE OF S100A9 IN MI

MI refers to a drastic reduction in myocardial blood and oxygen supply due to reduced or interrupted coronary blood flow, which subsequently induces ischemic necrosis of cardiac myocytes.^77^ Cardiomyocyte death is regulated by various complex processes in the early stages of MI.^77^ During the initial hours post‐MI, myocardial cells undergo swelling and necrosis, the interstitium of the myocardium becomes edematous and eosinophils infiltrate; in subsequent inflammatory phase, characterized by extensive infiltration of inflammatory cells (primarily neutrophils) into the necrotic area, activation of these inflammatory cells and death of cardiomyocytes leads to an elevation in the local and circulating concentrations of S100A9, which initiates an inflammatory cascade via triggering downstream signalling pathways and participating in the process of myocardial injury; concurrently, macrophages play a role from the inflammatory phase to the granulation tissue stage and are involved in both myocardial injury and repair processes.^78^, ^79^, ^80^, ^81^, ^82^, ^83^

Researchers have discovered numerous tissue peptides and proteins contributing to the diagnosis and prognosis of MI.^84^ Healy et al.^85^ employed a transcriptome analysis approach to compare the differential expression of platelet mRNA transcripts between patients with ST‐segment elevation MI (STEMI) and those with stable coronary artery disease. They identified that S100A9 is a novel regulator of thrombus formation and found that S100A9, one of the strongest predictors in the microarray data analysis (*p* = 0.002), exhibited increased expression prior to STEMI occurrence.^85^ This suggests that S100A9 is a candidate biomarker for predicting the onset of MI.

The study has shown that compared to individuals with stable CVD or normal individuals, patients with ACS exhibit local and systemic S100A8/A9 levels that exceed the critical threshold of 8.0 mg/L within 3 h of symptom onset, making it a promising candidate for detecting ACS.^86^ Notably, Fraccarollo et al.^87^ investigated the correlation between S100A9 and MI in 47 patients and found that MI patients exhibited high expression of S100A9 in circulating CD10^neg^ neutrophils compared to patients with stable angina. Furthermore, another study reported a significant elevation of S100A9 levels in platelets of MI patients, and the abundance of S100A9 in platelets showed a strong positive correlation with neutrophil count (*R* = 0.54, *p* = 0.0025), indicating that S100A9 in platelets originates from neutrophils.^88^ These studies collectively indicate a specific elevation of S100A9 in MI, highlighting its potential as a potential biomarker for MI diagnosis with promising clinical applications.^86^, ^87^, ^88^

The latest study reported that the application of LASSO regression and SVM‐RFE algorithms could identify 11 overlapping genes, and ROC analysis of these 11 overlapping genes in the training sets GSE48060 and GSE66360 revealed that the genes with the highest area under the curve (AUC) reconciliation mean contained S100A9; further simplification of the diagnostic model demonstrated that the genes with over 90% accuracy in the training set contained S100A9, indicating that S100A9 is an effective biomarker for diagnosing MI.^89^

S100A9 is associated with the prognosis of MI. Marinković et al.^80^ reported that patients with significantly elevated plasma S100A9 within 24 h after MI had an increased length of stay and an increased incidence of major adverse cardiovascular events due to heart failure during the follow‐up. Sreejit et al.^90^ and Li et al.^91^ further showed that patients with higher neutrophils level following ACS, which secret S100A9, had a significantly higher incidence of major adverse cardiovascular events during the 1‐year follow‐up after revascularization. These findings suggest that the detection of elevated expression of S100A9 in early MI indicates a poor prognosis, and S100A9 levels or neutrophil counts could predict MI prognosis.^80^, ^90^, ^91^

**FIGURE 4:**
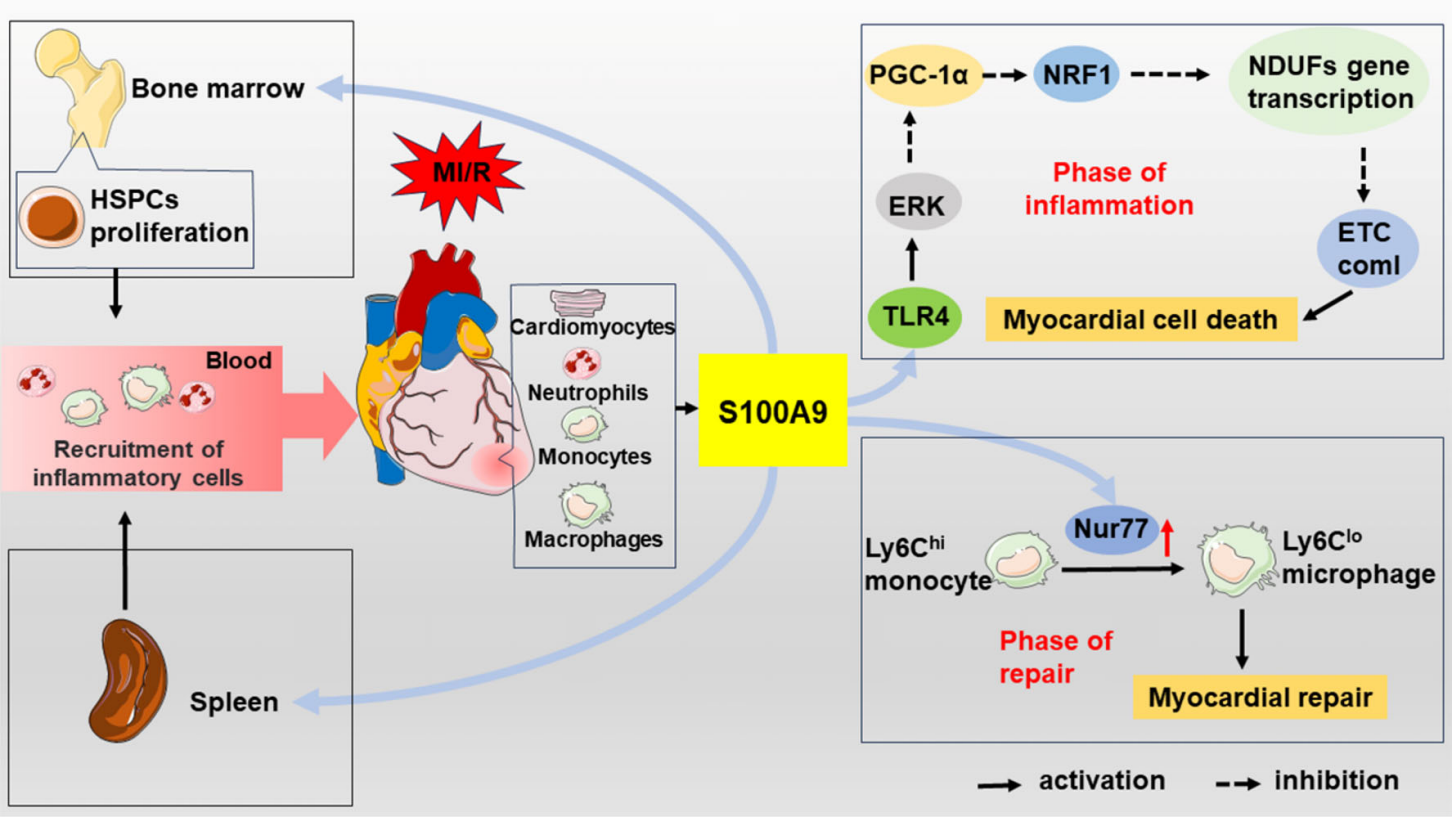
Role of the S100A9 in myocardial infarction. In MI, S100A9 promotes HSPCs proliferation and recruits inflammatory cells to the ischemic myocardium. Dying cardiomyocytes, neutrophils, monocytes, and macrophages further secrete S100A9. In the inflammatory phase, S100A9 binds to TLR4, activates ERK, downregulates PGC‐1α, inhibits NRF1, and impairs mitochondrial complex I, causing cardiomyocyte death. In the repair phase, S100A9 upregulates Nur77, facilitating the transition from Ly6C^hi^ monocytes to reparative Ly6C^lo^ macrophages and promoting myocardial repair. HSPCs, haematopoietic stem and progenitor cells; PGC‐1α, Pparg coactivator 1 alpha; NRF1, nuclear respiratory factor 1; NDUF, ETC complex I genes.

During the inflammatory phase of cardiac repair, S100A9 is mainly secreted by immune‐activated cells, resulting in a rapidly increasing S100A9 levels in the blood and heart, and a peak 3–5 days post‐MI.^19^, ^80^, ^91^, ^92^ Elevated S100A9 can stimulate the production of myeloid cells and translocation to ischemic myocardium, thereby promoting cardiac inflammation.^80^ Furthermore, thinning of the infarcted wall is significantly associated with increased expression of S100A9,^92^ which may contribute to post‐MI complications such as ventricular aneurysm due to weakening of the cardiac structure. Interestingly, during the proliferative phase post‐MI, S100A9 can mediate the transition from inflammatory Ly6C^hi^ monocytes to reparative Ly6C^lo^ macrophages by upregulating the levels and activity of Nur77 in macrophages and Ly6C^hi/int^ monocytes, which facilitates myocardial repair after injury^78^ (Figure 4).

Li et al.^91^ further investigated the impact of S100A9 in myocardial ischaemia–reperfusion (MI/R) and found that compared to WT mice, S100A9 knockout mice exhibited markedly reduced infarct size and improved cardiac function, while transgenic mice overexpressing S100A9 showed opposite results. The underlying mechanism involves the rescue of mitochondrial function upon S100A9 knockout, which is associated with the restoration of the TLR4/ERK‐mediated PGC‐1α/NRF1 signalling pathway.^91^ This restoration leads to the recovery of gene expression related to the mitochondrial complex I subunit NDUFs. Through these intricate processes, the study elucidates the specific mechanism behind early cardiomyocyte death in the context of MI, highlighting the pivotal pro‐inflammatory role played by S100A9 (Figure 4). The study confirms that S100A9 critically regulates cardiomyocyte death and survival by impacting mitochondrial function, making it a key mediator in the progression towards cardiomyocyte death.^91^

However, in models of MI induced by permanent coronary artery ligation, Marinković et al.^78^ observed that S100A9^−/−^ mice exhibited a decreased number of circulating monocytes and reduced infiltration of monocytes and macrophages into the heart on the 7th day after MI compared to WT mice, most importantly the numbers of repairing Ly6C^lo^MerTK^hi^ macrophages were reduced by approximately 50% in the myocardium of S100A9^−/−^ mice; on the 21st day after MI, S100A9^−/−^ mice showed worse cardiac function. These findings suggest that the positive impact of S100A9 on cardiac repair after MI is ineffective during the acute phase of MI; meanwhile, indicating that early blockade of S100A9 is beneficial for cardiac repair, while prolonging the blockade may have adverse effects.^78^, ^91^

## 5 ROLE OF S100A9 IN OTHER VASCULAR DISEASES

The pathological basis of vascular diseases is the narrowing or occlusion of the lumen of blood vessels due to multiple causes, leading to ischemic changes in organs or limbs, and some vascular diseases present local dilatation and tumour‐like changes.^93^, ^94^ Common clinical vascular diseases including PAH, aneurysm, PAD and aortic coarctation have complex and diverse aetiologies, in which inflammatory responses are involved in pathogenesis.^95^, ^96^, ^97^, ^98^ S100A9, a pro‐inflammatory alarm factor, is also involved in the developmental process of these vascular diseases.

PAH is a life‐threatening disease manifested by progressive pulmonary vascular remodelling and characterized by perivascular inflammatory infiltrates of varying degrees of inflammation, including macrophages, neutrophils, T cells, and B cells.^99^, ^100^, ^101^, ^102^ In addition to immune cells infiltration around the blood vessels, levels of cytokines such as S100A9, IL‐6, IL‐1β, and TNF‐α are also abnormally elevated.^102^, ^103^, ^104^ PAH has multifactorial aetiology and complex pathological mechanisms. Currently, inflammation, fibrosis, epigenetic factors, right ventricular dysfunction, and gender differences are all considered to be associated with the development of PAH, with chronic inflammation playing a significant role in the pathogenesis of the disease.^105^, ^106^, ^107^

Research has shown that the pro‐inflammatory mediator S100A9 is overexpressed in pulmonary arterial smooth muscle cells (PASMCs) of patients with PAH.^104^ Of note, Zeng et al.^108^ identified S100A9 as a promising biomarker for PAH using a combination of LASSO regression analysis and SVM‐RFE algorithm. Subsequent evaluation of the diagnostic value of S100A9 in PAH revealed an impressive AUC of 0.931 (95% CI = 0.832–0.981) in GSE117621 and an AUC of 0.722 (95% CI = 0.457–0.907) in GSE4819, underscoring the significance of S100A9 in PAH diagnosis.^108^ S100A9 activation is positively correlated with immune cells, including neutrophils, monocytes, and T cells, aligning with its role as an inflammatory mediator.^108^ Additionally, a differential gene expression analysis showed that S100A9 is consistently present in both SARS‐CoV‐2 and PAH samples, with a heat map indicating a high‐risk tendency propensity for S100A9 expression, implying an increased risk of PAH following SARS‐CoV‐2 infection.^109^ These findings highlight the crucial regulatory role of S100A9 in PAH‐related inflammation.

As classical receptors for S100A9, both RAGE and TLR4 demonstrate consistent upregulation in PAH. Compared to healthy individuals, PAH patients exhibit excessive expression of RAGE in their plasma, pulmonary arteries, PASMCs, endothelial cells, and fibroblasts.^104^, ^110^ This augmented expression of RAGE leads to the generation of pro‐inflammatory cytokines and cellular proliferation, playing a pivotal role in the pathogenesis and progression of PAH. Moreover, in circulating monocytes and PASMCs of PAH patients, both TLR4 mRNA and protein levels are significantly elevated.^111^, ^112^ Upregulation of TLR4 leads to increased PASMCs proliferation and decreased apoptosis, promoting vascular remodelling in PAH. This phenomenon is closely related to TLR4 being a target gene of miR‐503 and regulated by the LncRNA MALAT1, the latter of which has been demonstrated to play a crucial role in vascular remodelling.^111^ Importantly, the increased expression of TLR4 activates the classical NF‐κB inflammatory signalling pathway, thereby mediating endothelial cell inflammation within pulmonary arteries and emerging as one of the principal factors driving the pathogenesis of PAH.^112^

**FIGURE 5:**
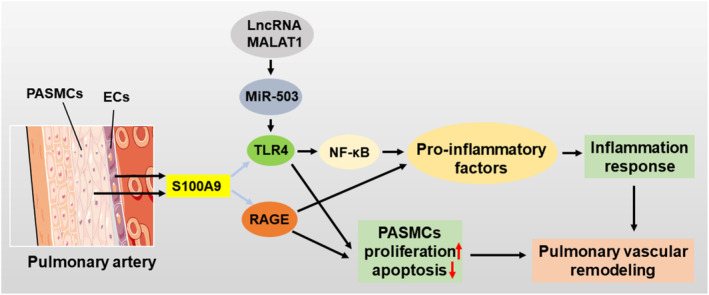
Possible mechanism of vascular remodelling induction by S100A9. The expression of S100A9, TLR4, and RAGE is increased in PASMCs and ECs, and thus S100A9 is hypothesized to contribute to pulmonary vascular remodelling by binding to TLR4/RAGE. ECs, endothelial cells; PASMCs, pulmonary artery smooth muscle cells; LncRNA MALAT1, long‐chain noncoding RNA metastasis‐related lung adenocarcinoma transcript 1; MiR‐503, microRNA‐503.

Considering the uniform upregulation of S100A9 and its classical receptors, RAGE and TLR4, in PAH, coupled with their ability to trigger inflammatory responses and perturb immune system homeostasis upon heightened expression, we speculate that the interplay between S100A9 and RAGE or TLR4 might constitute one of the underlying mechanisms driving the pathogenesis of PAH (Figure 5).

In other hypertensive disorders, S100A9 also plays a pivotal role. Preeclampsia stands out as a prevalent hypertensive disorder during pregnancy, with its pathogenesis involving maternal infections and inflammation.^113^ Recent investigation has revealed heightened levels of S100A9 in the blood plasma and placenta of pregnant women affected by preeclampsia.^114^ S100A9 triggers the activation of the NLRP3 inflammasome in both the placenta and human umbilical vein endothelial cells, leading to the secretion of IL‐1β and soluble endoglin.^114^ Moreover, the administration of exogenous S100A9 stimulates the secretion of soluble endoglin and the accumulation of neutrophils in pregnant mice, subsequently inducing maternal hypertension.^114^ These findings significantly imply that S100A9 potentially participates in the pathogenesis of pre‐eclampsia.^114^ Furthermore, a study has found that upon the initial day of angiotensin II infusion in mice, there is a substantial upregulation of S100A9 expression, which consequently contributes to the development of hypertension‐induced cardiac injury caused by angiotensin II.^24^ These compelling findings underscore the potential of both S100A9 as critical indicators for monitoring the occurrence and progression of hypertension.

Aneurysm is a significant vascular disease whose development is also associated with S100A9. It has shown that compared to *Porphyromonas gingivalis* negative subjects, positive subjects exhibited a significant increase in the number of aneurysms, and a notable overexpression of S100A9 was detected on the smooth muscle cells surface of damaged vessels, indicating that *P. gingivalis* might promote the occurrence and development of aortic aneurysms through upregulating S100A9.^115^ Additionally, Nakaoka et al.^116^ used gene expression profiling to reveal that the levels of S100A9 were significantly higher in ruptured aneurysms compared to unruptured intracranial aneurysms, and early stage ruptured aneurysms showed higher levels of S100A9 than late‐stage ruptured aneurysms. Recently, a clinical study involving 63 patients reported that the median concentration of S100A8/A9 in the venous blood of aneurysm patients was 1257 ng/ml, significantly exceeding the median S100A8/A9 concentration of 390 ng/ml in healthy individuals (*p* ≤ 0.001); the median concentration of S100A8/A9 in ruptured aneurysms was 8530 ng/ml, which was significantly surpassed in unruptured aneurysms (*p* = 0.04).^117^ These findings suggest that S100A9 could serve as a molecular biomarker for diagnosing and distinguishing aneurysms prone to rupture and as a potential intervention target.

S100A9 is not only a marker for the diagnosis and differentiation of aneurysms, but also an important substance for predicting the prognosis of aneurysms. Lech et al.^118^ found persistently elevated plasma S100A9 levels for months to decades in patients suffering from giant aneurysms after Kawasaki disease and monocytes that infiltrated the coronary arteries and cardiomyocytes continually expressed S100A9. Furthermore, a plasma S100A8/A9 concentration higher than 6020 pg/ml (sensitivity 53.57%, specificity 96.15%) within 48 h after the onset of aneurysmal subarachnoid haemorrhage is indicative of a poor prognosis.^119^ These demonstrate that S100A9 may be a marker for predicting the prognosis of aneurysms. Significantly, a recent study has reported an association between S100A9 and aortic dissection. Single‐cell sequencing technology revealed an elevated proportion of pro‐inflammatory macrophages within the arterial walls of patients suffering from aortic dissection, compared to the control group, with these macrophages demonstrating high expression of S100A9.^120^ This suggests that pro‐inflammatory macrophages may through the secretion of S100A9 mediate vascular inflammation, contributing to the development of aortic dissection.^120^ However, whether S100A9 serves as a target for the treatment of aneurysms and aortic dissection remains to be further investigated.

In PAD‐affected limbs, there has been confirmation of immune and inflammatory cell infiltration, with S100A9 showing elevated expression in these affected limbs.^121^ A recent report by Saenz‐Pipaon et al.^122^ revealed that S100A9 mRNA is abundant in extracellular vesicles and plasma of PAD patients and increased with the severity of PAD, suggesting S100A9 as a biomarker for predicting the severity of PAD. When S100A9 combines with the inflammatory marker hs‐CRP or lipocalin‐2, it best predicts the risk of amputation and major adverse cardiovascular events in PAD.^122^, ^123^ This indicates that the approach of multiple marker combination is of superior value in predicting PAD risk.

M1 macrophages, known as classically activated macrophages, mainly participate in pro‐inflammatory responses.^124^ A study has indicated that in preclinical PAD models, the increased expression of VEGF_165b_ in macrophages inhibits the phosphorylation of VEGFR1, leading to an increase in downstream S100A9, and an increase in calcium influx, thereby inducing MI‐like polarization of macrophages, ultimately inhibiting angiogenesis and reperfusion recovery in ischemic muscles of PAD.^125^ This confirms that targeting S100A9 may be beneficial for restoring blood flow in the affected limb.

## 6 ROLE OF S100A9 IN CARDIOMYOPATHY AND ATRIAL FIBRILLATION

In cardiomyopathy, alterations in cardiac structure and abnormalities in cardiac electrophysiology may precipitate the occurrence of atrial fibrillation (AF), and sustained AF can further aggravate cardiac remodelling, resulting in a progressive deterioration of cardiac function.^126^, ^127^, ^128^ Importantly, inflammatory reactions have closely implicated in hypertrophic cardiomyopathy (HCM), uremic cardiomyopathy (UCM), and AF.^129^, ^130^, ^131^

In HCM, Zhao et al.^132^ identified immune infiltration‐related genes through RNA sequencing, discovering that S100A9 is predominantly expressed by infiltrating M1 macrophages in the cardiac immune microenvironment, especially CCR2‐M1 macrophages, and also confirmed that S100A9 is a potential biomarker for differentiating HCM from controls. But its specific molecular mechanism remains to be further explored.

In addition, Cai et al.^130^ observed an upregulation in the transcriptional level of S100A9 in UCM rats. Furthermore, compared to UCM rats, the degree of myocardial cell hypertrophy and fibrosis was significantly improved in the UCM rats transduced with AAV‐shS100A9, as evidenced by decreased mRNA and protein levels of fibrosis markers (TGFb1, α‐SMA, collagen 4a1, and fibronectin) in myocardial tissue; and the expression of inflammatory cytokines (IL‐6, TNF‐α, and IL‐1β) in both the local myocardium and systemic circulation was reduced.^130^ These indicate that knockdown of S100A9 can alleviate the inflammatory response and tissue fibrosis in UCM, thereby improving cardiac function.^130^

AF is a common arrhythmia caused by various factors such as inflammation and atrial fibrosis.^131^ Liu et al.^133^ identified S100A9 as the most strongly associated factor with AF using the PPI network and LASSO model. Further analysis of the ROC curve determined the AUC of S100A9 in AF patients and controls.^133^ In the training cohort, the AUC was 0.9981 (95% CI, 0.993–1), while in the testing cohort, the AUC was 0.862 (95% CI, 0.7271–1), indicating the diagnostic value of S100A9 for AF and S100A9 is identifying as a potential AF biomarker.^133^ Chu et al.^134^ also showed a significant correlation between S100A9 and AF using univariate logistic regression analysis, while the results of the binomial logistic regression analysis from the generalized linear model demonstrated a monotonic relationship between the two, proving that the risk of AF increases with the increase in S100A9 gene expression. In light of the fact that atrial inflammation can lead to atrial electrical remodelling and structural changes, resulting in the onset of AF,^135^ and considering the observed positive correlation between S100A9 and AF,^133^, ^134^ we posit that the inflammatory response triggered by S100A9 may play a role in the pathogenesis of AF.

These studies have greatly enriched our understanding of the pro‐inflammatory effects of S100A9, highlighting its essential role as a significant endogenous damage‐associated molecular pattern. Moreover, these findings have provided compelling evidence supporting the potential significance of S100A9 as a key biomarker in cardiomyopathy and AF. Thus, the assessment and intervention of S100A9 expression hold promising value for the diagnosis and treatment of these specific disorders.

## 7 ROLE OF S100A9 IN OTHER INFECTION‐ASSOCIATED CVDs

Myocarditis is an important inflammatory disease of the myocardium, commonly associated with Coxsackie virus B (CVB) infection. Müller et al.^62^ reported a 5.1‐fold increase (*p* = 0.038) in S100A9 expression in the endocardial myocardial tissues of myocarditis patients infected with CVB3 compared to the control group. Conversely, a decrease in serum S100A9 levels was associated with reduced cardiac inflammation, suggesting that low S100A9 levels may indicate a favourable prognosis for myocarditis.^136^ In experimental research, increased expression of S100A9 mRNA and protein levels were likewise found in the heart and spleen of mice with myocarditis compared to control mice.^137^ Upregulation of S100A9 leads to increased secretion of its downstream pro‐inflammatory cytokines, which in turn leads to myocardial inflammation, an effect that has been associated with increased cardiac viral load due to increased cardiac myeloid‐derived suppressor cell counts.^137^ Furthermore, compared with myocarditis mice, the mRNA levels of RAGE and Dia‐1 were significantly reduced in the hearts of CVB3‐infected S100A9^−/−^ mice, while there was no statistical difference in MyD88, indicating that CVB3 may induce myocarditis by activating the S100A9‐RAGE‐Dia‐1 pathway, and defective S100A9 gene leads to the suppression of this axis thereby attenuating myocardial inflammation.^62^

Currently, there is limited research exploring the connection between infective endocarditis (IE) and S100A9. Nonetheless, Xiao et al.^138^ identified S100A9 as a key gene involved in IE through bioinformatics analysis and found that S100A9 plays a crucial role in the inflammation and immune response of IE, suggesting that S100A9 may be involved in the pathogenesis of IE.

## 8 PERSPECTIVES ON THE CLINICAL APPLICATION OF S100A9 AS A THERAPEUTIC TARGET

**FIGURE 6:**
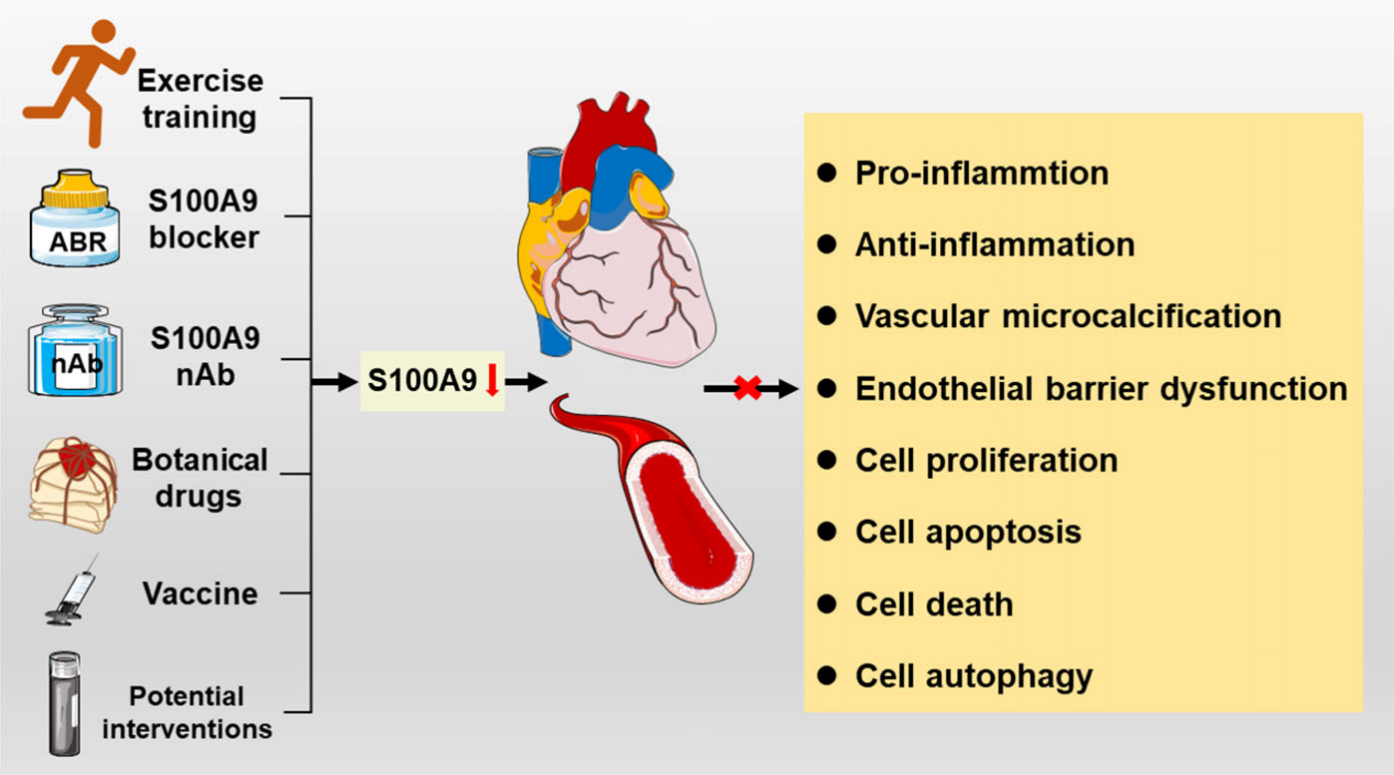
S100A9's diverse impact on inflammation and cardiovascular health. S100A9 exhibits a dual regulatory role in the inflammatory response. It can induce endothelial cell dysfunction, promote vascular calcification, and facilitate cell proliferation, autophagy, apoptosis, and death. However, these effects can be mitigated by various interventions, including exercise training, ABR, S100A9 nAb, botanical drugs, vaccines against S100A9 protein, and potential interventions such as siRNA therapy. S100A9 nAb, S100A9 neutralizing antibody.

In CVD, S100A9 plays a regulatory role in inflammation, calcium balance, endothelial function, cell proliferation, autophagy, apoptosis, and cell death. Currently, S100A9 inhibitors, such as ABR‐215757 (Paquinimod), ABR‐215062 (Laquinimod), and ABR‐215050 (Tasquinimod) have been used in various stages of clinical trials, and given the increased expression of S100A9 in the development of CVD; these inhibitors have potential for the treatment of CVD as well. Furthermore, S100A9 neutralizing antibodies (nAb), botanical drugs, exercise training, and active vaccination against S100A9 have been confirmed to have cardiovascular protective effects (Figure 6).

Paquinimod and ABR‐238901 primarily exert their effects by blocking the interaction between S100A9 and TLR4 and RAGE.^76^, ^139^ Paquinimod has demonstrated affirmative efficacy and safety in phase II clinical trials for systemic sclerosis.^140^ Laquinimod, a quinoline‐3‐carboxamide, exhibits binding capability to S100A9, yet its precise mechanism of action remains to be elucidated.^23^ Some studies propose that laquinimod mainly functions through the reduction of NF‐κB, direct inhibition of T cells, and modification of antigen‐presenting cell subpopulations.^141^, ^142^ Laquinimod has been subjected to phase III clinical trials for multiple sclerosis and phase II clinical trials for Crohn's disease, manifesting promising therapeutic outcomes.^143^, ^144^ Tasquinimod, another S100A9 inhibitor, predominantly exerts its effects by blocking the interaction between S100A9 and TLR4.^145^ Although it has demonstrated progression‐free survival during phase III clinical trials for prostate cancer, the anticipated effects were not observed in phase II studies for advanced hepatocellular carcinoma, gastric cancer, kidney cancer, and ovarian cancer.^146^, ^147^ All of these drugs mentioned above are small molecule immunomodulators that work by affecting immune cells and inflammatory processes. While they have not yet been applied to clinical trials for CVD, their extensive application in animal models underscores their potential for future clinical trials and subsequent clinical therapeutic applications.

Marinković et al.^80^ reported that intraperitoneal injection of ABR‐238901 into mice with MI for three consecutive days (at the onset of MI, 24 h post‐MI, and 48 h post‐MI) significantly improve ejection fraction and cardiac output. However, 21 days of treatment lead to progressive deterioration of cardiac function and ventricular remodelling.^78^ It is indicated that short‐term blockade of S100A9 can ameliorate post‐MI inflammatory injury and promote cardiac repair, while extended treatment duration can lead to the opposite effect, correlating with a reduction in reparative macrophages due to long‐term S100A9 blockade. Interestingly, compared to the control group, S100A9 dimer pretreatment enhances the anti‐inflammatory effect of human amniotic mesenchymal stem cells at days 7, 14, and 28 post‐MI, facilitating the recovery of cardiac function.^50^ These findings reveal the dual pro‐inflammatory and anti‐inflammatory roles of S100A9 in the post‐MI repair process, emphasizing the crucial importance of identifying the time window for blocking S100A9 for MI prognosis.

In addition, Li et al.^91^ have developed an S100A9 nAb that has a neutralizing effect both in vitro and in vivo. In MI mice, this nAb can significantly reduce the infarct area, upregulate the activity of mitochondrial complex I, alleviate cardiac fibrosis and improve cardiac function.^91^ Nevertheless, the clinical application of this antibody remains pending, and its safety and efficacy as a viable therapeutic option necessitate further investigation and clinical trials.

Studies show that botanical drugs protect the heart by inhibiting macrophage activation, reducing the release of S100A9, and suppressing the expression of inflammatory factors such as IL‐1β and TNF‐α.^25^, ^148^ It is known that exercise training plays an important role in cardiac rehabilitation. Reports suggest that exercise can stimulate macrophages in heart failure mice to secrete IL‐10, promote p‐STAT3/S100A9 nuclear translocation, and regulate the differentiation of myeloid‐derived suppressor cells, thus achieving cardioprotection.^149^, ^150^ It is worth noting that S100A9 nAb, botanical drugs, and exercise exert beneficial effects in CVD by reducing S100A9, further emphasizing S100A9 as a potential intervention target.

The tobacco mosaic virus (TMV) is an RNA virus that can bind to the S100A9 targeting peptide. If TMV can be conjugated with drugs, then utilizing S100A9‐targeted TMV nanoparticles might assist in the development of new methods for clinical diagnosis and treatment of CVD.^151^ Notably, vaccines against S100A9 have shown antithrombotic and anti‐atherosclerotic treatments in animal models, and this class of vaccines will be a new strategy for preventing the worsening or recurrence of CVD due to poor medication adherence.^152^, ^153^, ^154^ And with the continuous advancement of nanotechnology, the delivery efficiency of siRNA is expected to be significantly improved,^155^, ^156^ providing new methods and perspectives for targeting S100A9. Therefore, S100A9 represents a crucial target for treating CVD.

## 9 CONCLUSION

Inflammation serves as a fundamental initiator for various cardiovascular pathologies, and studies have revealed that monitoring immune‐inflammatory responses holds paramount significance in diagnosing, predicting, and treating CVD. S100A9, a pivotal inflammatory modulator, has been reported to participate in the development and progression of diverse cardiovascular disorders. According to its physiological characteristics and mechanisms of action, S100A9 is prominently engaged in the regulation of inflammatory responses (including pro‐inflammation and anti‐inflammation), endothelial function, cellular proliferation, autophagy, apoptosis, and cell death. Through these intricate pathways, S100A9 assumes a pivotal role in driving the onset and advancement of CVD. Conversely, studies have revealed that the inhibition of S100A9 holds pivotal therapeutic significance in a plethora of CVD, encompassing atherosclerosis, MI, PAH, PAD, cardiomyopathy, and myocarditis. S100A9 inhibitors have already been employed as clinic trial medications for various inflammation‐related disorders, implying their potential application value in CVD. However, considering the complexity of S100A9's actions and the unique characteristics of inflammatory responses in CVD, further research is warranted at different stages and with diverse formulations to better understand its role.

## AUTHOR CONTRIBUTIONS

The design of study was done by YG and ZC, manuscript was drafted by FC and revised by YG, ZH, CW, and JS. All authors read and approved the final manuscript.

## CONFLICT OF INTEREST STATEMENT

The authors have no conflicts of interest to disclose.

## References

[1] Joseph P , Leong D , McKee M , et al. Reducing the global burden of cardiovascular disease, part 1: the epidemiology and risk factors. Circ Res. 2017;121(6):677‐694. doi:10.1161/CIRCRESAHA.117.308903 28860318

[2] Schultz WM , Kelli HM , Lisko JC , et al. Socioeconomic status and cardiovascular outcomes: challenges and interventions. Circulation. 2018;137(20):2166‐2178. doi:10.1161/CIRCULATIONAHA.117.029652 29760227 PMC5958918

[3] Imig JD , Cervenka L , Neckar J . Epoxylipids and soluble epoxide hydrolase in heart diseases. Biochem Pharmacol. 2022;195:114866. doi:10.1016/j.bcp.2021.114866 34863976 PMC8712413

[4] Golia E , Limongelli G , Natale F , et al. Inflammation and cardiovascular disease: from pathogenesis to therapeutic target. Curr Atheroscler Rep. 2014;16(9):435. doi:10.1007/s11883-014-0435-z 25037581

[5] Speer T , Dimmeler S , Schunk SJ , Fliser D , Ridker PM . Targeting innate immunity‐driven inflammation in CKD and cardiovascular disease. Nat Rev Nephrol. 2022;18(12):762‐778. doi:10.1038/s41581-022-00621-9 36064794

[6] Yi X , Zhu QX , Wu XL , Tan TT , Jiang XJ . Histone methylation and oxidative stress in cardiovascular diseases. Oxid Med Cell Longev. 2022;2022:6023710. doi:10.1155/2022/6023710 35340204 PMC8942669

[7] Sorriento D , Iaccarino G . Inflammation and cardiovascular diseases: the Most recent findings. Int J Mol Sci. 2019;20(16):3879. doi:10.3390/ijms20163879 31395800 PMC6719998

[8] Mohebi R , McCarthy CP , Gaggin HK , van Kimmenade RRJ , Januzzi JL . Inflammatory biomarkers and risk of cardiovascular events in patients undergoing coronary angiography. Am Heart J. 2022;252:51‐59. doi:10.1016/j.ahj.2022.06.004 35753356 PMC9336200

[9] Travers JG , Tharp CA , Rubino M , McKinsey TA . Therapeutic targets for cardiac fibrosis: from old school to next‐gen. J Clin Invest. 2022;132(5):e148554. doi:10.1172/JCI148554 35229727 PMC8884906

[10] Guo Y , Chen J , Qiu H . Novel mechanisms of exercise‐induced cardioprotective factors in myocardial infarction. Front Physiol. 2020;11:199. doi:10.3389/fphys.2020.00199 32210839 PMC7076164

[11] Senoner T , Dichtl W . Oxidative stress in cardiovascular diseases: still a therapeutic target? Nutrients. 2019;11(9):2090. doi:10.3390/nu11092090 31487802 PMC6769522

[12] Medina‐Leyte DJ , Zepeda‐García O , Domínguez‐Pérez M , González‐Garrido A , Villarreal‐Molina T , Jacobo‐Albavera L . Endothelial dysfunction, inflammation and coronary artery disease: potential biomarkers and promising therapeutical approaches. Int J Mol Sci. 2021;22(8):3850. doi:10.3390/ijms22083850 33917744 PMC8068178

[13] Jin Z , Niu J , Kapoor N , Liang J , Becerra E , Kolattukudy PE . Essential role of endothelial MCPIP in vascular integrity and post‐ischemic remodeling. Int J Mol Sci. 2019;20(1):172. doi:10.3390/ijms20010172 30621250 PMC6337340

[14] Xu S , Ilyas I , Little PJ , et al. Endothelial dysfunction in atherosclerotic cardiovascular diseases and beyond: from mechanism to pharmacotherapies. Pharmacol Rev. 2021;73(3):924‐967. doi:10.1124/pharmrev.120.000096 34088867

[15] Immanuel J , Yun S . Vascular inflammatory diseases and endothelial phenotypes. Cells. 2023;12(12):1640. doi:10.3390/cells12121640 37371110 PMC10297687

[16] Zheng Y , Huang S , Zhang J , et al. Melatonin alleviates vascular endothelial cell damage by regulating an autophagy‐apoptosis axis in Kawasaki disease. Cell Prolif. 2022;55(6):e13251. doi:10.1111/cpr.13251 35582751 PMC9201377

[17] Silvestre‐Roig C , Braster Q , Ortega‐Gomez A , Soehnlein O . Neutrophils as regulators of cardiovascular inflammation. Nat Rev Cardiol. 2020;17(6):327‐340. doi:10.1038/s41569-019-0326-7 31996800

[18] Wang S , Song R , Wang Z , Jing Z , Wang S , Ma J . S100A8/A9 in inflammation. Front Immunol. 2018;9:1298. doi:10.3389/fimmu.2018.01298 29942307 PMC6004386

[19] Schiopu A , Cotoi OS . S100A8 and S100A9: DAMPs at the crossroads between innate immunity, traditional risk factors, and cardiovascular disease. Mediators Inflamm. 2013;2013:828354. doi:10.1155/2013/828354 24453429 PMC3881579

[20] Chen X , He J , Xie Y , et al. Tetrahedral framework nucleic acid nanomaterials reduce the inflammatory damage in sepsis by inhibiting pyroptosis. Cell Prolif. 2023;56(8):e13424. doi:10.1111/cpr.13424 36802079 PMC10392044

[21] Bhardwaj RS , Zotz C , Roth J , et al. The calcium‐binding proteins MRP8 and MRP14 form a membrane‐associated heterodimer in a subset of monocytes/macrophages present in acute but absent in chronic inflammatory lesions. Eur J Immunol. 1992;22(7):1891‐1897. doi:10.1002/eji.1830220732 1378023

[22] Hunter MJ , Chazin WJ . High level expression and dimer characterization of the S100 EF‐hand proteins, migration inhibitory factor‐related proteins 8 and 14. J Biol Chem. 1998;273(20):12427‐12435. doi:10.1074/jbc.273.20.12427 9575199

[23] Björk P , Björk A , Vogl T , et al. Identification of human S100A9 as a novel target for treatment of autoimmune disease via binding to quinoline‐3‐carboxamides. PLoS Biol. 2009;7(4):e1000097. doi:10.1371/journal.pbio.1000097 19402754 PMC2671563

[24] Wu Y , Li Y , Zhang C , et al. S100a8/a9 released by CD11b+Gr1+ neutrophils activates cardiac fibroblasts to initiate angiotensin II‐induced cardiac inflammation and injury. Hypertension. 2014;63(6):1241‐1250. doi:10.1161/HYPERTENSIONAHA.113.02843 24711518

[25] Sun Y , Wang Z , Hou J , et al. Shuangxinfang prevents S100A9‐induced macrophage/microglial inflammation to improve cardiac function and depression‐like behavior in rats after acute myocardial infarction. Front Pharmacol. 2022;13:832590. doi:10.3389/fphar.2022.832590 35814253 PMC9263923

[26] Simard JC , Cesaro A , Chapeton‐Montes J , et al. S100A8 and S100A9 induce cytokine expression and regulate the NLRP3 inflammasome via ROS‐dependent activation of NF‐κB(1.). PloS One. 2013;8(8):e72138. doi:10.1371/journal.pone.0072138 23977231 PMC3747084

[27] Zhang W , Lavine KJ , Epelman S , et al. Necrotic myocardial cells release damage‐associated molecular patterns that provoke fibroblast activation in vitro and trigger myocardial inflammation and fibrosis in vivo. J Am Heart Assoc. 2015;4(6):e001993. doi:10.1161/JAHA.115.001993 26037082 PMC4599537

[28] Xia C , Braunstein Z , Toomey AC , Zhong J , Rao X . S100 proteins As an important regulator of macrophage inflammation. Front Immunol. 2017;8:1908. doi:10.3389/fimmu.2017.01908 29379499 PMC5770888

[29] Fan S , Zhao H , Liu Y , et al. Isoproterenol triggers ROS/P53/S100‐A9 positive feedback to aggravate myocardial damage associated with complement activation. Chem Res Toxicol. 2020;33(10):2675‐2685. doi:10.1021/acs.chemrestox.0c00308 32924446

[30] Wang L , Luo H , Chen X , Jiang Y , Huang Q . Functional characterization of S100A8 and S100A9 in altering monolayer permeability of human umbilical endothelial cells. PloS One. 2014;9(3):e90472. doi:10.1371/journal.pone.0090472 24595267 PMC3940892

[31] Dahlem C , Kado SY , He Y , et al. AHR signaling interacting with nutritional factors regulating the expression of markers in vascular inflammation and atherogenesis. Int J Mol Sci. 2020;21(21):E8287. doi:10.3390/ijms21218287 PMC7663825 33167400

[32] Zhao B , Yu J , Luo Y , et al. Deficiency of S100 calcium binding protein A9 attenuates vascular dysfunction in aged mice. Redox Biol. 2023;63:102721. doi:10.1016/j.redox.2023.102721 37163872 PMC10189516

[33] Nakanishi T , Iida S , Maruyama J , et al. Arteriosclerosis derived from cutaneous inflammation is ameliorated by the deletion of IL‐17A and IL‐17F. Int J Mol Sci. 2023;24(6):5434. doi:10.3390/ijms24065434 36982506 PMC10049365

[34] Liu Y , Luo G , He D . Clinical importance of S100A9 in osteosarcoma development and as a diagnostic marker and therapeutic target. Bioengineered. 2019;10(1):133‐141. doi:10.1080/21655979.2019.1607709 31055998 PMC6527076

[35] Itou H , Yao M , Fujita I , et al. The crystal structure of human MRP14 (S100A9), a Ca(2+)‐dependent regulator protein in inflammatory process. J Mol Biol. 2002;316(2):265‐276. doi:10.1006/jmbi.2001.5340 11851337

[36] Salminen A , Vlachopoulou E , Havulinna AS , et al. Genetic variants contributing to circulating matrix metalloproteinase 8 levels and their association with cardiovascular diseases: a genome‐wide analysis. Circ Cardiovasc Genet. 2017;10(6):e001731. doi:10.1161/CIRCGENETICS.117.001731 29212897

[37] Chen H , Lunney JK , Cheng L , et al. Porcine S100A8 and S100A9: molecular characterizations and crucial functions in response to *Haemophilus parasuis* infection. Dev Comp Immunol. 2011;35(4):490‐500. doi:10.1016/j.dci.2010.11.017 21185856

[38] Tamulytė R , Jankaitytė E , Toleikis Z , Smirnovas V , Jankunec M . Pro‐inflammatory protein S100A9 alters membrane organization by dispersing ordered domains. Biochim Biophys Acta Biomembr. 2023;1865(3):184113. doi:10.1016/j.bbamem.2022.184113 36567033

[39] Pruenster M , Immler R , Roth J , et al. E‐selectin‐mediated rapid NLRP3 inflammasome activation regulates S100A8/S100A9 release from neutrophils via transient gasdermin D pore formation. Nat Immunol. 2023;30:2021‐2031. doi:10.1038/s41590-023-01656-1 PMC10681899 37903858

[40] Fan ZP , Peng ML , Chen YY , et al. S100A9 activates the immunosuppressive switch through the PI3K/Akt pathway to maintain the immune suppression function of testicular macrophages. Front Immunol. 2021;12:743354. doi:10.3389/fimmu.2021.743354 34764959 PMC8576360

[41] Srikrishna G . S100A8 and S100A9: New insights into their roles in malignancy. J Innate Immun. 2011;4(1):31‐40. doi:10.1159/000330095 21912088 PMC3250655

[42] Averill MM , Barnhart S , Becker L , et al. S100A9 differentially modifies phenotypic states of neutrophils, macrophages, and dendritic cells: implications for atherosclerosis and adipose tissue inflammation. Circulation. 2011;123(11):1216‐1226. doi:10.1161/CIRCULATIONAHA.110.985523 21382888 PMC3072335

[43] Monteiro C , Miarka L , Perea‐García M , et al. Stratification of radiosensitive brain metastases based on an actionable S100A9/RAGE resistance mechanism. Nat Med. 2022;28(4):752‐765. doi:10.1038/s41591-022-01749-8 35411077 PMC9018424

[44] Xiao X , Yang C , Qu SL , et al. S100 proteins in atherosclerosis. Clin Chim Acta. 2020;502:293‐304. doi:10.1016/j.cca.2019.11.019 31794767

[45] Bertolini I , Perego M , Nefedova Y , et al. Intercellular hif1α reprograms mammary progenitors and myeloid immune evasion to drive high‐risk breast lesions. J Clin Invest. 2023;133(8):e164348. doi:10.1172/JCI164348 36892943 PMC10104898

[46] Huang X , Shen W , Veizades S , Liang G , Sayed N , Nguyen PK . Single‐cell transcriptional profiling reveals sex and age diversity of gene expression in mouse endothelial cells. Front Genet. 2021;12:590377. doi:10.3389/fgene.2021.590377 33679877 PMC7929607

[47] Boteanu RM , Suica VI , Uyy E , et al. Short‐term blockade of pro‐inflammatory alarmin S100A9 favorably modulates left ventricle proteome and related signaling pathways involved in post‐myocardial infarction recovery. Int J Mol Sci. 2022;23(9):5289. doi:10.3390/ijms23095289 35563680 PMC9103348

[48] Mihaila AC , Ciortan L , Macarie RD , et al. Transcriptional profiling and functional analysis of N1/N2 neutrophils reveal an immunomodulatory effect of S100A9‐blockade on the pro‐inflammatory N1 subpopulation. Front Immunol. 2021;12:708770. doi:10.3389/fimmu.2021.708770 34447377 PMC8384118

[49] Ursino G , Lucibello G , Teixeira PDS , et al. S100A9 exerts insulin‐independent antidiabetic and anti‐inflammatory effects. Sci Adv. 2024;10(1):eadj4686. doi:10.1126/sciadv.adj4686 38170783 PMC10796079

[50] Chen TJ , Yeh YT , Peng FS , Li AH , Wu SC . S100A8/A9 enhances immunomodulatory and tissue‐repairing properties of human amniotic mesenchymal stem cells in myocardial ischemia‐reperfusion injury. Int J Mol Sci. 2021;22(20):11175. doi:10.3390/ijms222011175 34681835 PMC8541313

[51] Vogl T , Stratis A , Wixler V , et al. Autoinhibitory regulation of S100A8/S100A9 alarmin activity locally restricts sterile inflammation. J Clin Invest. 2018;128(5):1852‐1866. doi:10.1172/JCI89867 29611822 PMC5919817

[52] Zhan X , Wu R , Kong XH , et al. Elevated neutrophil extracellular traps by HBV‐mediated S100A9‐TLR4/RAGE‐ROS cascade facilitate the growth and metastasis of hepatocellular carcinoma. Cancer Commun. 2023;43(2):225‐245. doi:10.1002/cac2.12388 PMC9926958 36346061

[53] Zhang X , Wei L , Wang J , et al. Suppression colitis and colitis‐associated colon cancer by anti‐S100a9 antibody in mice. Front Immunol. 2017;8:1774. doi:10.3389/fimmu.2017.01774 29326691 PMC5733461

[54] Li C , Chen H , Ding F , et al. A novel p53 target gene, S100A9, induces p53‐dependent cellular apoptosis and mediates the p53 apoptosis pathway. Biochem J. 2009;422(2):363‐372. doi:10.1042/BJ20090465 19534726

[55] Zhang Y , Zha Z , Shen W , et al. Anemoside B4 ameliorates TNBS‐induced colitis through S100A9/MAPK/NF‐κB signaling pathway. Chin Med. 2021;16(1):11. doi:10.1186/s13020-020-00410-1 33461587 PMC7814617

[56] Guo S , Su Q , Wen J , et al. S100A9 induces nucleus pulposus cell degeneration through activation of the NF‐κB signaling pathway. J Cell Mol Med. 2021;25(10):4709‐4720. doi:10.1111/jcmm.16424 33734570 PMC8107097

[57] Nagareddy PR , Kraakman M , Masters SL , et al. Adipose tissue macrophages promote myelopoiesis and monocytosis in obesity. Cell Metab. 2014;19(5):821‐835. doi:10.1016/j.cmet.2014.03.029 24807222 PMC4048939

[58] Ursino G , Ramadori G , Höfler A , et al. Hepatic non‐parenchymal S100A9‐TLR4‐mTORC1 axis normalizes diabetic ketogenesis. Nat Commun. 2022;13(1):4107. doi:10.1038/s41467-022-31803-5 35840613 PMC9287425

[59] Wang A , Guo B , Jia Q , Chen YU , Gao X , Xu S . S100A9‐containing serum exosomes of burn injury patients promote permeability of pulmonary microvascular endothelial cells. J Biosci. 2021;46:33. 33859068

[60] Lee NR , Park BS , Kim SY , et al. Cytokine secreted by S100A9 via TLR4 in monocytes delays neutrophil apoptosis by inhibition of caspase 9/3 pathway. Cytokine. 2016;86:53‐63. doi:10.1016/j.cyto.2016.07.005 27459393

[61] Yi W , Zhu R , Hou X , Wu F , Feng R . Integrated analysis reveals S100a8/a9 regulates autophagy and apoptosis through the MAPK and PI3K‐AKT signaling pathway in the early stage of myocardial infarction. Cells. 2022;11(12):1911. doi:10.3390/cells11121911 35741040 PMC9221389

[62] Müller I , Vogl T , Pappritz K , et al. Pathogenic role of the damage‐associated molecular patterns S100A8 and S100A9 in coxsackievirus B3‐induced myocarditis. Circ Heart Fail. 2017;10(11):e004125. doi:10.1161/CIRCHEARTFAILURE.117.004125 29158436

[63] Kawakami R , Katsuki S , Travers R , et al. S100A9‐RAGE Axis accelerates formation of macrophage‐mediated extracellular vesicle microcalcification in diabetes mellitus. Arterioscler Thromb Vasc Biol. 2020;40(8):1838‐1853. doi:10.1161/ATVBAHA.118.314087 32460581 PMC7377960

[64] Biswas AK , Han S , Tai Y , et al. Targeting S100A9‐ALDH1A1‐retinoic acid signaling to suppress brain relapse in EGFR‐mutant lung cancer. Cancer Discov. 2022;12(4):1002‐1021. doi:10.1158/2159-8290.CD-21-0910 35078784 PMC8983473

[65] Zha H , Li X , Sun H , et al. S100A9 promotes the proliferation and migration of cervical cancer cells by inducing epithelial‐mesenchymal transition and activating the Wnt/β‐catenin pathway. Int J Oncol. 2019;55(1):35‐44. doi:10.3892/ijo.2019.4793 31059008 PMC6561615

[66] Tumurkhuu G , Shimada K , Dagvadorj J , et al. Ogg1‐dependent DNA repair regulates NLRP3 inflammasome and prevents atherosclerosis. Circ Res. 2016;119(6):e76‐e90. doi:10.1161/CIRCRESAHA.116.308362 27384322 PMC5010464

[67] Kong P , Cui ZY , Huang XF , Zhang DD , Guo RJ , Han M . Inflammation and atherosclerosis: signaling pathways and therapeutic intervention. Signal Transduct Target Ther. 2022;7(1):131. doi:10.1038/s41392-022-00955-7 35459215 PMC9033871

[68] Zhaolin Z , Guohua L , Shiyuan W , Zuo W . Role of pyroptosis in cardiovascular disease. Cell Prolif. 2019;52(2):e12563. doi:10.1111/cpr.12563 30525268 PMC6496801

[69] Hansson GK , Hermansson A . The immune system in atherosclerosis. Nat Immunol. 2011;12(3):204‐212. doi:10.1038/ni.2001 21321594

[70] Libby P . The changing landscape of atherosclerosis. Nature. 2021;592(7855):524‐533. doi:10.1038/s41586-021-03392-8 33883728

[71] New SEP , Goettsch C , Aikawa M , et al. Macrophage‐derived matrix vesicles: an alternative novel mechanism for microcalcification in atherosclerotic plaques. Circ Res. 2013;113(1):72‐77. doi:10.1161/CIRCRESAHA.113.301036 23616621 PMC3703850

[72] Ionita MG , Vink A , Dijke IE , et al. High levels of myeloid‐related protein 14 in human atherosclerotic plaques correlate with the characteristics of rupture‐prone lesions. Arterioscler Thromb Vasc Biol. 2009;29(8):1220‐1227. doi:10.1161/ATVBAHA.109.190314 19520974

[73] Langley SR , Willeit K , Didangelos A , et al. Extracellular matrix proteomics identifies molecular signature of symptomatic carotid plaques. J Clin Invest. 2017;127(4):1546‐1560. doi:10.1172/JCI86924 28319050 PMC5373893

[74] Flynn MC , Kraakman MJ , Tikellis C , et al. Transient intermittent hyperglycemia accelerates atherosclerosis by promoting myelopoiesis. Circ Res. 2020;127(7):877‐892. doi:10.1161/CIRCRESAHA.120.316653 32564710 PMC7486277

[75] Hanssen NMJ , Tikellis C , Pickering RJ , et al. Pyridoxamine prevents increased atherosclerosis by intermittent methylglyoxal spikes in the aortic arches of ApoE−/− mice. Biomed Pharmacother. 2023;158:114211. doi:10.1016/j.biopha.2022.114211 36916437

[76] Kraakman MJ , Lee MK , Al‐Sharea A , et al. Neutrophil‐derived S100 calcium‐binding proteins A8/A9 promote reticulated thrombocytosis and atherogenesis in diabetes. J Clin Invest. 2017;127(6):2133‐2147. doi:10.1172/JCI92450 28504650 PMC5451242

[77] Thygesen K , Alpert JS , Jaffe AS , et al. Fourth universal definition of myocardial infarction. J Am Coll Cardiol. 2018;72(18):2231‐2264. doi:10.1016/j.jacc.2018.08.1038 30153967

[78] Marinković G , Koenis DS , de Camp L , et al. S100A9 links inflammation and repair in myocardial infarction. Circ Res. 2020;127(5):664‐676. doi:10.1161/CIRCRESAHA.120.315865 32434457

[79] Michaud K , Basso C , d'Amati G , et al. Diagnosis of myocardial infarction at autopsy: AECVP reappraisal in the light of the current clinical classification. Virchows Arch. 2020;476(2):179‐194. doi:10.1007/s00428-019-02662-1 31522288 PMC7028821

[80] Marinković G , Grauen Larsen H , Yndigegn T , et al. Inhibition of pro‐inflammatory myeloid cell responses by short‐term S100A9 blockade improves cardiac function after myocardial infarction. Eur Heart J. 2019;40(32):2713‐2723. doi:10.1093/eurheartj/ehz461 31292614

[81] Sreejit G , Nooti SK , Jaggers RM , et al. Retention of the NLRP3 inflammasome‐primed neutrophils in the bone marrow is essential for myocardial infarction‐induced granulopoiesis. Circulation. 2022;145(1):31‐44. doi:10.1161/CIRCULATIONAHA.121.056019 34788059 PMC8716427

[82] Pan W , Zhang J , Zhang L , et al. Comprehensive view of macrophage autophagy and its application in cardiovascular diseases. Cell Prolif. 2023;57:e13525. doi:10.1111/cpr.13525 37434325 PMC10771119

[83] Guo Y , Luo F , Liu Q , Xu D . Regulatory non‐coding RNAs in acute myocardial infarction. J Cell Mol Med. 2017;21(5):1013‐1023. doi:10.1111/jcmm.13032 27878945 PMC5387171

[84] Aydin S , Ugur K , Aydin S , Sahin İ , Yardim M . Biomarkers in acute myocardial infarction: current perspectives. Vasc Health Risk Manag. 2019;15:1‐10. doi:10.2147/VHRM.S166157 30697054 PMC6340361

[85] Healy AM , Pickard MD , Pradhan AD , et al. Platelet expression profiling and clinical validation of myeloid‐related protein‐14 as a novel determinant of cardiovascular events. Circulation. 2006;113(19):2278‐2284. doi:10.1161/CIRCULATIONAHA.105.607333 16682612

[86] Altwegg LA , Neidhart M , Hersberger M , et al. Myeloid‐related protein 8/14 complex is released by monocytes and granulocytes at the site of coronary occlusion: a novel, early, and sensitive marker of acute coronary syndromes. Eur Heart J. 2007;28(8):941‐948. doi:10.1093/eurheartj/ehm078 17387139

[87] Fraccarollo D , Neuser J , Möller J , Riehle C , Galuppo P , Bauersachs J . Expansion of CD10neg neutrophils and CD14+HLA‐DRneg/low monocytes driving proinflammatory responses in patients with acute myocardial infarction. Elife. 2021;10:e66808. doi:10.7554/eLife.66808 34289931 PMC8324297

[88] Joshi A , Schmidt LE , Burnap SA , et al. Neutrophil‐derived protein S100A8/A9 alters the platelet proteome in acute myocardial infarction and is associated with changes in platelet reactivity. Arterioscler Thromb Vasc Biol. 2022;42(1):49‐62. doi:10.1161/ATVBAHA.121.317113 34809447 PMC8691374

[89] Lin ZL , Liu YC , Gao YL , et al. S100A9 and SOCS3 as diagnostic biomarkers of acute myocardial infarction and their association with immune infiltration. Genes Genet Syst. 2022;97(2):67‐79. doi:10.1266/ggs.21-00073 35675985

[90] Sreejit G , Abdel‐Latif A , Athmanathan B , et al. Neutrophil‐derived S100A8/A9 amplify granulopoiesis after myocardial infarction. Circulation. 2020;141(13):1080‐1094. doi:10.1161/CIRCULATIONAHA.119.043833 31941367 PMC7122461

[91] Li Y , Chen B , Yang X , et al. S100a8/a9 signaling causes mitochondrial dysfunction and cardiomyocyte death in response to ischemic/reperfusion injury. Circulation. 2019;140(9):751‐764. doi:10.1161/CIRCULATIONAHA.118.039262 31220942

[92] Chalise U , Becirovic‐Agic M , Daseke MJ , et al. S100A9 is a functional effector of infarct wall thinning after myocardial infarction. Am J Physiol Heart Circ Physiol. 2022;322(2):H145‐H155. doi:10.1152/ajpheart.00475.2021 34890276 PMC8742737

[93] Simon F , Oberhuber A , Floros N , et al. Acute limb ischemia‐much more than just a lack of oxygen. Int J Mol Sci. 2018;19(2):374. doi:10.3390/ijms19020374 29373539 PMC5855596

[94] Richards GHC , Hong KL , Henein MY , Hanratty C , Boles U . Coronary artery ectasia: review of the non‐atherosclerotic molecular and pathophysiologic concepts. Int J Mol Sci. 2022;23(9):5195. doi:10.3390/ijms23095195 35563583 PMC9103542

[95] Hu Y , Chi L , Kuebler WM , Goldenberg NM . Perivascular inflammation in pulmonary arterial hypertension. Cells. 2020;9(11):2338. doi:10.3390/cells9112338 33105588 PMC7690279

[96] Cui H , Chen Y , Li K , et al. Untargeted metabolomics identifies succinate as a biomarker and therapeutic target in aortic aneurysm and dissection. Eur Heart J. 2021;42(42):4373‐4385. doi:10.1093/eurheartj/ehab605 34534287 PMC11506060

[97] Aday AW , Matsushita K . Epidemiology of peripheral artery disease and polyvascular disease. Circ Res. 2021;128(12):1818‐1832. doi:10.1161/CIRCRESAHA.121.318535 34110907 PMC8202714

[98] Chan NC , Xu K , de Vries TAC , Eikelboom JW , Hirsh J . Inflammation as a mechanism and therapeutic target in peripheral artery disease. Can J Cardiol. 2022;38(5):588‐600. doi:10.1016/j.cjca.2022.01.026 35114347

[99] Zhang L , Wang Y , Wu G , et al. Blockade of JAK2 protects mice against hypoxia‐induced pulmonary arterial hypertension by repressing pulmonary arterial smooth muscle cell proliferation. Cell Prolif. 2020;53(2):e12742. doi:10.1111/cpr.12742 31943454 PMC7046303

[100] Zhang Q , Cao Y , Luo Q , et al. The transient receptor potential vanilloid‐3 regulates hypoxia‐mediated pulmonary artery smooth muscle cells proliferation via PI3K/AKT signaling pathway. Cell Prolif. 2018;51(3):e12436. doi:10.1111/cpr.12436 29359496 PMC6528857

[101] Qiu H , Zhang Y , Li Z , et al. Donepezil ameliorates pulmonary arterial hypertension by inhibiting M2‐macrophage activation. Front Cardiovasc Med. 2021;8:639541. doi:10.3389/fcvm.2021.639541 33791350 PMC8005547

[102] Rabinovitch M , Guignabert C , Humbert M , Nicolls MR . Inflammation and immunity in the pathogenesis of pulmonary arterial hypertension. Circ Res. 2014;115(1):165‐175. doi:10.1161/CIRCRESAHA.113.301141 24951765 PMC4097142

[103] Zhang L , Zeng XX , Li YM , et al. Keratin 1 attenuates hypoxic pulmonary artery hypertension by suppressing pulmonary artery media smooth muscle expansion. Acta Physiol (Oxf). 2021;231(2):e13558. doi:10.1111/apha.13558 32920982

[104] Nakamura K , Sakaguchi M , Matsubara H , et al. Crucial role of RAGE in inappropriate increase of smooth muscle cells from patients with pulmonary arterial hypertension. PloS One. 2018;13(9):e0203046. doi:10.1371/journal.pone.0203046 30180189 PMC6122782

[105] Thenappan T , Ormiston ML , Ryan JJ , Archer SL . Pulmonary arterial hypertension: pathogenesis and clinical management. BMJ. 2018;360:j5492. doi:10.1136/bmj.j5492 29540357 PMC6889979

[106] Yaku A , Inagaki T , Asano R , et al. Regnase‐1 prevents pulmonary arterial hypertension through mRNA degradation of interleukin‐6 and platelet‐derived growth factor in alveolar macrophages. Circulation. 2022;146(13):1006‐1022. doi:10.1161/CIRCULATIONAHA.122.059435 35997026

[107] Guo Y , He Z , Chen Z , et al. Inhibition of Th17 cells by donepezil ameliorates experimental lung fibrosis and pulmonary hypertension. Theranostics. 2023;13(6):1826‐1842. doi:10.7150/thno.82069 37064881 PMC10091879

[108] Zeng H , Liu X , Zhang Y . Identification of potential biomarkers and immune infiltration characteristics in idiopathic pulmonary arterial hypertension using bioinformatics analysis. Front Cardiovasc Med. 2021;8:624714. doi:10.3389/fcvm.2021.624714 33598484 PMC7882500

[109] Taz TA , Ahmed K , Paul BK , Al‐Zahrani FA , Mahmud SMH , Moni MA . Identification of biomarkers and pathways for the SARS‐CoV‐2 infections that make complexities in pulmonary arterial hypertension patients. Brief Bioinform. 2021;22(2):1451‐1465. doi:10.1093/bib/bbab026 33611340 PMC7929374

[110] Diekmann F , Chouvarine P , Sallmon H , et al. Soluble receptor for advanced glycation end products (sRAGE) is a sensitive biomarker in human pulmonary arterial hypertension. Int J Mol Sci. 2021;22(16):8591. doi:10.3390/ijms22168591 34445297 PMC8395319

[111] He M , Shen J , Zhang C , Chen Y , Wang W , Tao K . Long‐chain non‐coding RNA metastasis‐related lung adenocarcinoma transcript 1 (MALAT1) promotes the proliferation and migration of human pulmonary artery smooth muscle cells (hPASMCs) by regulating the MicroRNA‐503 (miR‐503)/toll‐like receptor 4 (TLR4) signal axis. Med Sci Monit. 2020;26:e923123. doi:10.12659/MSM.923123 32712618 PMC7377003

[112] Zuo ZT , Ma Y , Sun Y , Bai CQ , Zhou HY , Chen BH . Role of TLR4/NF‐κB signalling pathway in pulmonary arterial hypertension in patients with chronic obstructive pulmonary disease. J Coll Physicians Surg Pak. 2020;30(6):568‐573. doi:10.29271/jcpsp.2020.06.568 32703338

[113] Jung E , Romero R , Yeo L , et al. The etiology of preeclampsia. Am J Obstet Gynecol. 2022;226(2S):S844‐S866. doi:10.1016/j.ajog.2021.11.1356 35177222 PMC8988238

[114] Ozeki A , Oogaki Y , Henmi Y , et al. Elevated S100A9 in preeclampsia induces soluble endoglin and IL‐1β secretion and hypertension via the NLRP3 inflammasome. J Hypertens. 2022;40(1):84‐93. doi:10.1097/HJH.0000000000002981 34412079

[115] Nakano K , Wada K , Nomura R , et al. Characterization of aortic aneurysms in cardiovascular disease patients harboring *Porphyromonas gingivalis* . Oral Dis. 2011;17(4):370‐378. doi:10.1111/j.1601-0825.2010.01759.x 21029263

[116] Nakaoka H , Tajima A , Yoneyama T , et al. Gene expression profiling reveals distinct molecular signatures associated with the rupture of intracranial aneurysm. Stroke. 2014;45(8):2239‐2245. doi:10.1161/STROKEAHA.114.005851 24938844

[117] de Korte AM , Aquarius R , Vogl T , et al. Elevation of inflammatory S100A8/S100A9 complexes in intracranial aneurysms. J Neurointerv Surg. 2020;12(11):1117‐1121. doi:10.1136/neurintsurg-2019-015753 32332055

[118] Lech M , Guess J , Duffner J , et al. Circulating markers of inflammation persist in children and adults with giant aneurysms after Kawasaki disease. Circ Genom Precis Med. 2019;12(4):e002433. doi:10.1161/CIRCGEN.118.002433 30844302

[119] Wang C , Kou Y , Han Y , Li X . Early serum calprotectin (S100A8/A9) predicts delayed cerebral ischemia and outcomes after aneurysmal subarachnoid hemorrhage. J Stroke Cerebrovasc Dis. 2020;29(5):104770. doi:10.1016/j.jstrokecerebrovasdis.2020.104770 32173226

[120] Zhang B , Zeng K , Guan RC , et al. Single‐cell RNA‐seq analysis reveals macrophages are involved in the pathogenesis of human sporadic acute type A aortic dissection. Biomolecules. 2023;13(2):399. doi:10.3390/biom13020399 36830768 PMC9952989

[121] Salyers ZR , Mariani V , Balestrieri N , et al. S100A8 and S100A9 are elevated in chronically threatened ischemic limb muscle and induce ischemic mitochondrial pathology in mice. JVS Vasc Sci. 2022;3:232‐245. doi:10.1016/j.jvssci.2022.03.003 35647565 PMC9133641

[122] Saenz‐Pipaon G , San Martín P , Planell N , et al. Functional and transcriptomic analysis of extracellular vesicles identifies calprotectin as a new prognostic marker in peripheral arterial disease (PAD). J Extracell Vesicles. 2020;9(1):1729646. doi:10.1080/20013078.2020.1729646 32158521 PMC7048174

[123] Saenz‐Pipaon G , Ravassa S , Larsen KL , et al. Lipocalin‐2 and calprotectin potential prognosis biomarkers in peripheral arterial disease. Eur J Vasc Endovasc Surg. 2022;63(4):648‐656. doi:10.1016/j.ejvs.2022.01.012 35307155

[124] Li J , Yao Y , Wang Y , et al. Modulation of the crosstalk between Schwann cells and macrophages for nerve regeneration: a therapeutic strategy based on a multifunctional tetrahedral framework nucleic acids system. Adv Mater. 2022;34(46):e2202513. doi:10.1002/adma.202202513 35483031

[125] Ganta VC , Choi M , Farber CR , Annex BH . Antiangiogenic VEGF165b regulates macrophage polarization via S100A8/S100A9 in peripheral artery disease. Circulation. 2019;139(2):226‐242. doi:10.1161/CIRCULATIONAHA.118.034165 30586702 PMC6322929

[126] Maron BJ . Clinical course and Management of Hypertrophic Cardiomyopathy. N Engl J Med. 2018;379(7):655‐668. doi:10.1056/NEJMra1710575 30110588

[127] Garg L , Gupta M , Sabzwari SRA , et al. Atrial fibrillation in hypertrophic cardiomyopathy: prevalence, clinical impact, and management. Heart Fail Rev. 2019;24(2):189‐197. doi:10.1007/s10741-018-9752-6 30456592

[128] Lekawanvijit S . Cardiotoxicity of uremic toxins: a driver of cardiorenal syndrome. Toxins. 2018;10(9):352. doi:10.3390/toxins10090352 30200452 PMC6162485

[129] Becker RC , Owens AP , Sadayappan S . Tissue‐level inflammation and ventricular remodeling in hypertrophic cardiomyopathy. J Thromb Thrombolysis. 2020;49(2):177‐183. doi:10.1007/s11239-019-02026-1 31898271 PMC7001758

[130] Cai X , Hong L , Liu Y , Huang X , Lai H , Shao L . Salmonella pathogenicity island 1 knockdown confers protection against myocardial fibrosis and inflammation in uremic cardiomyopathy via down‐regulation of S100 calcium binding protein A8/A9 transcription. Ren Fail. 2022;44(1):1819‐1832. doi:10.1080/0886022X.2022.2137421 36299239 PMC9621201

[131] Ajoolabady A , Nattel S , Lip GYH , Ren J . Inflammasome signaling in atrial fibrillation: JACC state‐of‐the‐art review. J Am Coll Cardiol. 2022;79(23):2349‐2366. doi:10.1016/j.jacc.2022.03.379 35680186 PMC8972346

[132] Zhao W , Wu T , Zhan J , Dong Z . Identification of the immune status of hypertrophic cardiomyopathy by integrated analysis of bulk‐ and single‐cell RNA sequencing data. Comput Math Methods Med. 2022;2022:7153491. doi:10.1155/2022/7153491 36238494 PMC9553329

[133] Liu L , Yu Y , Hu LL , et al. Potential target genes in the development of atrial fibrillation: a comprehensive bioinformatics analysis. Med Sci Monit. 2021;27:e928366. doi:10.12659/MSM.928366 33741890 PMC7989062

[134] Chu Y , Yu F , Wu Y , et al. Identification of genes and key pathways underlying the pathophysiological association between nonalcoholic fatty liver disease and atrial fibrillation. BMC Med Genomics. 2022;15(1):150. doi:10.1186/s12920-022-01300-1 35790963 PMC9258143

[135] Ihara K , Sasano T . Role of inflammation in the pathogenesis of atrial fibrillation. Front Physiol. 2022;13:862164. doi:10.3389/fphys.2022.862164 35492601 PMC9047861

[136] Müller I , Vogl T , Kühl U , et al. Serum alarmin S100A8/S100A9 levels and its potential role as biomarker in myocarditis. ESC Heart Fail. 2020;7(4):1442‐1451. doi:10.1002/ehf2.12760 32462801 PMC7373886

[137] Müller I , Janson L , Sauter M , et al. Myeloid‐derived suppressor cells restrain natural killer cell activity in acute coxsackievirus B3‐induced myocarditis. Viruses. 2021;13(5):889. doi:10.3390/v13050889 34065891 PMC8151145

[138] Xiao SJ , Zhou YF , Jia H , Wu Q , Pan DF . Identification of the pivotal differentially expressed genes and pathways involved in Staphylococcus aureus‐induced infective endocarditis by using bioinformatics analysis. Eur Rev Med Pharmacol Sci. 2021;25(1):487‐497. doi:10.26355/eurrev_202101_24420 33506940

[139] Schiopu A , Marinkovic G , De Camp L , et al. Short‐term blockade of the S100A8/A9 alarmin in the immediate post‐myocardial infarction period inhibits acute myocardial inflammation and preserves myocardial repair. Eur Heart J. 2017;38(suppl_1):ehx504.P4026. doi:10.1093/eurheartj/ehx504.P4026

[140] Hesselstrand R , Distler JHW , Riemekasten G , et al. An open‐label study to evaluate biomarkers and safety in systemic sclerosis patients treated with paquinimod. Arthritis Res Ther. 2021;23(1):204. doi:10.1186/s13075-021-02573-0 34330322 PMC8325221

[141] Brück W , Pförtner R , Pham T , et al. Reduced astrocytic NF‐κB activation by laquinimod protects from cuprizone‐induced demyelination. Acta Neuropathol. 2012;124(3):411‐424. doi:10.1007/s00401-012-1009-1 22766690 PMC3422618

[142] Schulze‐Topphoff U , Shetty A , Varrin‐Doyer M , et al. Laquinimod, a quinoline‐3‐carboxamide, induces type II myeloid cells that modulate central nervous system autoimmunity. PloS One. 2012;7(3):e33797. doi:10.1371/journal.pone.0033797 22479444 PMC3316495

[143] Comi G , Dadon Y , Sasson N , et al. CONCERTO: a randomized, placebo‐controlled trial of oral laquinimod in relapsing‐remitting multiple sclerosis. Mult Scler. 2022;28(4):608‐619. doi:10.1177/13524585211032803 34378456

[144] D'Haens G , Sandborn WJ , Colombel JF , et al. A phase II study of laquinimod in Crohn's disease. Gut. 2015;64(8):1227‐1235. doi:10.1136/gutjnl-2014-307118 25281416 PMC4515993

[145] Du Y , Cai Y , Lv Y , et al. Single‐cell RNA sequencing unveils the communications between malignant T and myeloid cells contributing to tumor growth and immunosuppression in cutaneous T‐cell lymphoma. Cancer Lett. 2022;551:215972. doi:10.1016/j.canlet.2022.215972 36265653

[146] Armstrong AJ , Anand A , Edenbrandt L , et al. Phase 3 assessment of the automated bone scan index as a prognostic imaging biomarker of overall survival in men with metastatic castration‐resistant prostate cancer: a secondary analysis of a randomized clinical trial. JAMA Oncol. 2018;4(7):944‐951. doi:10.1001/jamaoncol.2018.1093 29799999 PMC6145727

[147] Escudier B , Faivre S , Van Cutsem E , et al. A phase II multicentre, open‐label, proof‐of‐concept study of Tasquinimod in hepatocellular, ovarian, renal cell, and gastric cancers. Target Oncol. 2017;12(5):655‐661. doi:10.1007/s11523-017-0525-2 28798986

[148] Sun Y , Wang Z , Wang C , Tang Z , Zhao H . Psycho‐cardiology therapeutic effects of Shuangxinfang in rats with depression‐behavior post acute myocardial infarction: focus on protein S100A9 from proteomics. Biomed Pharmacother. 2021;144:112303. doi:10.1016/j.biopha.2021.112303 34673424

[149] Zhou L , Miao K , Yin B , et al. Cardioprotective role of myeloid‐derived suppressor cells in heart failure. Circulation. 2018;138(2):181‐197. doi:10.1161/CIRCULATIONAHA.117.030811 29437117

[150] Feng L , Li G , An J , et al. Exercise training protects against heart failure via expansion of myeloid‐derived suppressor cells through regulating IL‐10/STAT3/S100A9 pathway. Circ Heart Fail. 2022;15(3):e008550. doi:10.1161/CIRCHEARTFAILURE.121.008550 34911348

[151] Park J , Gao H , Wang Y , Hu H , Simon DI , Steinmetz NF . S100A9‐targeted tobacco mosaic virus nanoparticles exhibit high specificity toward atherosclerotic lesions in ApoE‐/‐ mice. J Mater Chem B. 2019;7(11):1842‐1846. doi:10.1039/c8tb02276c 32255046 PMC7147689

[152] Kawano T , Shimamura M , Nakagami H , et al. Therapeutic vaccine against S100A9 (S100 calcium‐binding protein A9) inhibits thrombosis without increasing the risk of bleeding in ischemic stroke in mice. Hypertension. 2018;72(6):1355‐1364. doi:10.1161/HYPERTENSIONAHA.118.11316 30571223

[153] Shimamura M , Kaikita K , Nakagami H , et al. Development of anti‐thrombotic vaccine against human S100A9 in rhesus monkey. Sci Rep. 2021;11(1):11472. doi:10.1038/s41598-021-91153-y 34075153 PMC8169762

[154] Ortega‐Rivera OA , Shin MD , Moreno‐Gonzalez MA , Pokorski JK , Steinmetz NF . A single‐dose Qβ VLP vaccine against S100A9 protein reduces atherosclerosis in a preclinical model. Adv Ther. 2022;5(10):2200092. doi:10.1002/adtp.202200092 PMC9783282 36570039

[155] Gao Y , Chen X , Tian T , et al. A lysosome‐activated tetrahedral nanobox for encapsulated siRNA delivery. Adv Mater. 2022;34(46):e2201731. doi:10.1002/adma.202201731 35511782

[156] Tian T , Li Y , Lin Y . Prospects and challenges of dynamic DNA nanostructures in biomedical applications. Bone Res. 2022;10(1):40. doi:10.1038/s41413-022-00212-1 35606345 PMC9125017

